# Covalent Organic Framework Electrolytes for Solid‐State Lithium Batteries: Designing Ion Highways for Next‐Generation Energy Storage

**DOI:** 10.1002/smll.75286

**Published:** 2026-08-25

**Authors:** Rajesh Dhanushkotti, Ningaraju G. N., Karthik C. S., Karthik V, Anil Kumar M R, Jitendrasingh Rajpurohit, Jeremy I. G. Dawkins, Karim Zaghib

**Affiliations:** ^1^ Department of Chemical and Materials Engineering Concordia University Montreal Quebec Canada; ^2^ Department of Chemistry SJCE JSS Science and Technology University Mysuru Karnataka India

**Keywords:** covalent organic frameworks, ion highways, lithium‐ion transport, nanochannels, solid‐state electrolytes, structure–property correlations

## Abstract

Covalent organic frameworks (COFs) are emerging as a programmable platform for solid electrolytes in solid‐state lithium batteries, since their crystalline, periodically ordered nanochannels can be engineered to enable directional ion transport. These low‐resistance pathways promote directional lithium‐ion (Li^+^) migration through a narrower, more spatially ordered distribution of coordination environments, achieved via site‐to‐site hopping and/or guest‐assisted vehicular motion within the channels. This review presents a design‐oriented framework linking COF structural descriptors to ion‐transport metrics through a cause‐effect‐performance relationship. We first summarize the Li^+^ transport mechanism in confined COF channels, covering site‐to‐site hopping along pore walls and guest‐assisted vehicular motion in filled channels. We then correlate (i) topology and channel architecture, (ii) pore‐wall chemical functionality and framework charge, (iii) crystallinity, stacking registry, and channel alignment, and (iv) hybrid COF architectures with performance indicators including ionic conductivity (σ), activation energy (Ea), lithium‐ion transference number (tLi^+^), transport anisotropy, and effective conductivity in working membranes (σeff). Through representative case studies, we highlight cases where high intrinsic channel transport does and does not translate into device‐relevant σeff. Finally, we propose standardized reporting practices and device‐level benchmarks to accelerate the shift from empirical materials discovery toward predictive ion‐highway engineering for next‐generation solid‐state lithium batteries.

## Graphical abstract

Covalent organic frameworks provide a structurally programmable platform for solid‐state lithium batteries. This review outlines a design framework linking structural descriptors to ion‐transport metrics. It examines mechanisms of confinement, channel architecture, and hybrid designs, addressing the reasons for the occasional failure of high intrinsic conductivity in operational membranes to guide advancements in energy storage solutions.

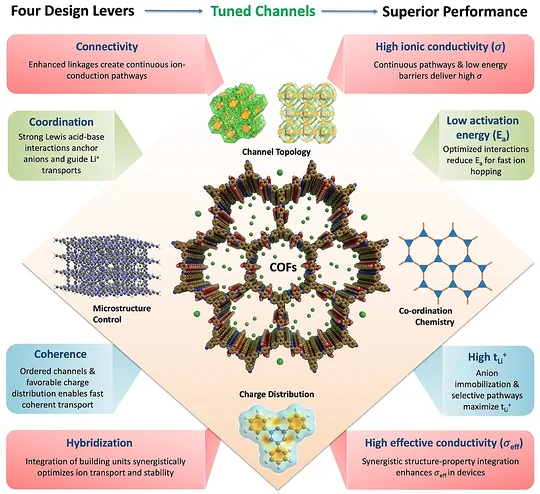

## 1 Introduction

The rapid development of sustainable energy technologies, including electric vehicles and large‐scale grid storage systems, is primarily driven by progress in electrochemical energy storage [1]. While the commercialization of lithium‐ion batteries (LIBs) has fundamentally changed the future of portable electronics, this widely adopted technology still faces two critical and interconnected limitations that hinder its application in next‐generation systems: safety issues and limited energy density [2]. Traditional LIBs use highly flammable organic liquid electrolytes that are susceptible to thermal runaway and pose inherent fire hazards [3]. At the same time, the requirement of long driving ranges and high energy outputs requires the use of the lithium (Li) metal anode, which has the highest theoretical specific capacity (3860 mAh g^−1^) and lowest electrochemical potential, but which is inherently incompatible with the conventional lithium liquid electrolyte systems [4]. During repeated cycling, the liquid electrolyte readily decomposes, facilitating the formation of Li dendrites, needle‐like crystalline structures that grow uncontrollably and penetrate the separator of the battery, leading to internal short circuits and thermal failure [5, 6].

The solution to these challenges is the development of all‐solid‐state lithium batteries (ASSLBs), in which the volatile liquid medium is replaced by a solid‐state electrolyte (SSE) [7, 8]. An ideal SSE must therefore satisfy a strict set of requirements simultaneously: it should exhibit ionic conductivity comparable to that of liquid electrolytes (typically σ_Li+_ > 10^−4^ S cm^−1^ at room temperature), possess excellent mechanical strength (to suppress Li dendrites physically), maintain a wide electrochemical stability window (ESW), and most importantly, display a high lithium‐ion transference number (t_Li+_ approaching 1) [9]. A high t_Li+_ ensures that almost all the charge is carried by the desired Li^+^ ion, thereby reducing concentration polarisation and enhancing power capability, which is largely difficult to achieve with conventional polymer or liquid electrolytes, in which both the cation (Li^+^) and the anion (PF_6_^−^ or TFSI^−^) are highly mobile [10].

Historically, research on SSEs has focused on two dominant classes of materials. Inorganic solid electrolytes (e.g., garnet‐type Li_7_La_3_Zr_2_O_12_ [11] or sulfide‐based LGPS‐type conductors [12] are capable of offering high intrinsic ionic conductivity. Still, their brittleness and processing limitations typically lead to imperfect electrode contact and high contact resistance, particularly under the volume changes during lithium‐metal cycling. In contrast, solid polymer electrolytes (SPEs), especially poly (ethylene oxide) (PEO)‐based systems, offer mechanical flexibility and conformal electrode contact. Furthermore, Li^+^ migration remains associated with slow segmental motion, resulting in inadequate conductivity at or near room temperature. Consequently, most solid‐state lithium batteries are still limited by the difficulty of simultaneously achieving high conductivity, mechanical robustness, and stable electrode contact within an electrolyte architecture [13, 14, 15, 16]. Figure 1 provides an overview of the rationale for this review, from the challenges facing present battery technologies to the opportunities offered by COF‐based electrolytes.

**FIGURE 1:**
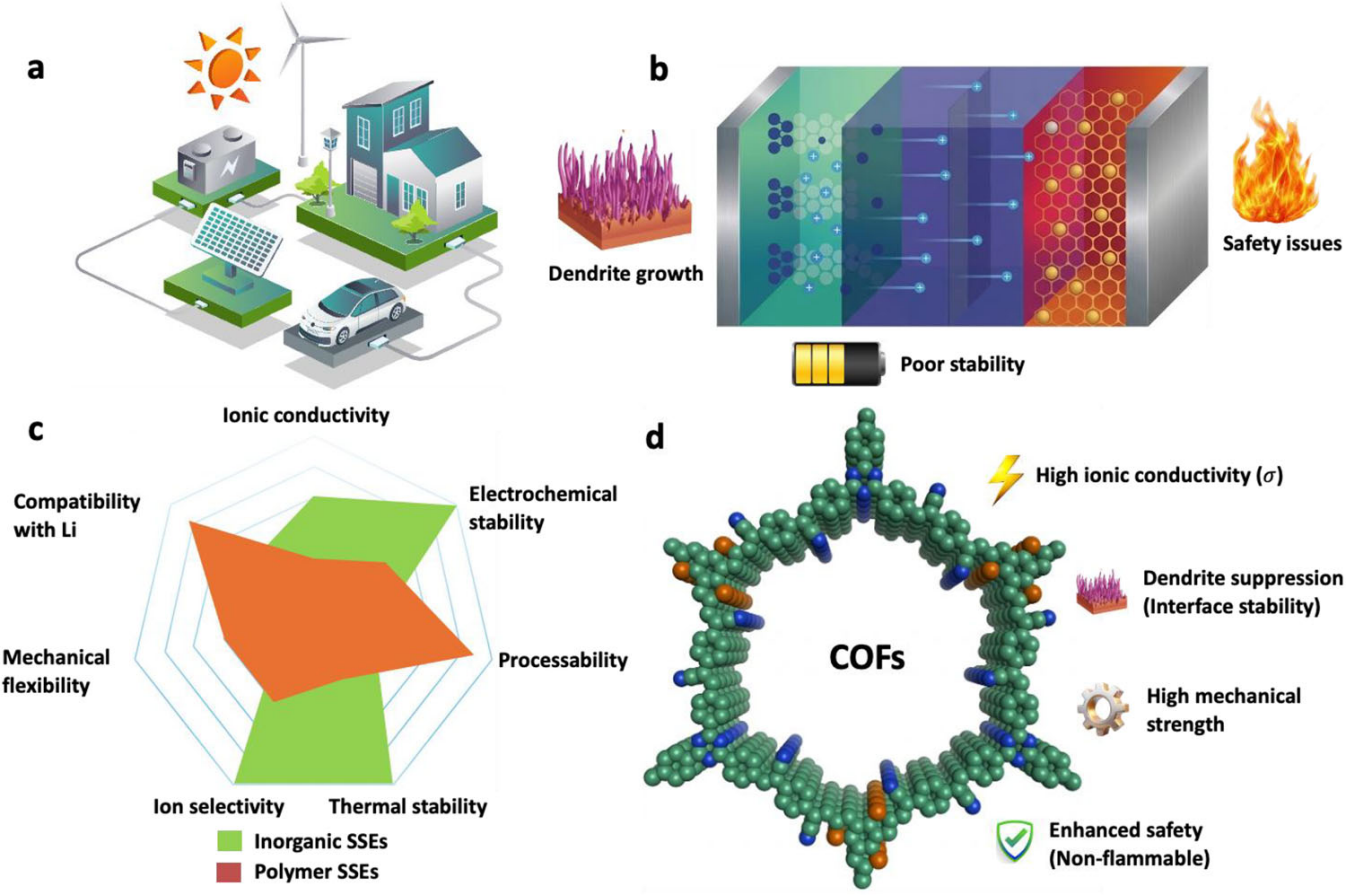
Overview of (a) renewable‑energy demand and storage needs, (b) safety and performance limits of current Li‑ion batteries, (c) structural and transport features of solid electrolytes, and (d) ordered COF channels enabling high ionic conductivity and non‑flammability.

This critical performance gap has driven the emergence of hybrid and highly ordered organic materials, most notably Covalent Organic Frameworks (COFs). COFs are a special class of crystalline, porous, organic polymer materials synthesized by the reversible condensation of small molecular building units connected by strong, directional covalent bonds (e.g., imine, boronate ester, or β‐ketoenamine) [14]. This ordered molecular structure contrasts sharply with the random, amorphous structure of traditional polymers, which instead form periodic, stable networks. Their defining attributes, specifically, permanent porosity, high surface area, and extraordinary chemical and thermal stability, have made COFs highly attractive components for the development of advanced electrochemical devices [15]. Importantly, stacked 2D COFs can, under favorable stacking registry and sufficient domain coherence, generate quasi‐1D nanochannels along the stacking direction [16]. In practical materials, the presence of slipped/turbostratic disorder and finite crystalline domain size can break the axial continuity of these channels. They should therefore be regarded as a design target: when coherence and texture/alignment are maintained, they provide structurally pre‐defined pathways—Ion Highways—for the directed migration of Li^+^, outperforming disordered polymer hopping once device‐scale percolating pathways are expressed in oriented/densified membranes [17].

Previous comprehensive reviews in the field have played an important role in summarizing the fundamental synthetic breakthroughs and outlining the applications of COFs, validating their role as a versatile platform in energy storage and spanning roles as electrode active materials, separators, and conductive fillers. Among these developments, particular attention has recently turned to the fabrication of redox‐active covalent organic frameworks and structurally related covalent organic polymers, which have demonstrated viable performance when integrated into lithium‐ion electrochemical systems [18, 19, 20]. For electrolyte systems, the current literature strongly emphasizes the empirical observation that incorporating COFs often improves ionic conductivity and Li^+^ transport in composite systems. However, a pervasive and limiting challenge remains: the notable lack of robust, quantitative, and predictive design rules that can translate these fundamental observations into intentional, performance‐driven engineering strategies [21].

The success noted in many studies often seems retrospective. At the same time, while the benefits of COFs’ incorporation in terms of the resulting properties are widely recognized, the exact atomistic and chemical principles that govern the complex host–guest interactions between the framework's pore walls, the mobile Li^+^ ion, and the mobile anion have not yet been unified into a cohesive, predictive framework. Consequently, the field has accumulated substantial data on COF properties (e.g., porosity, stability). Yet it has struggled to develop general guidelines for designing a COF structure that optimizes Li^+^ transport kinetics while simultaneously minimizing parasitic effects [22].

This review aims to address this knowledge gap by proposing a single, design‐forward paradigm for COF‐based solid‐state electrolytes that engineer ion highways. Within this framework, COFs are considered transport architectures whose performance arises from the coupling of four controllable levers: (i) connectivity, characterized by topology, pore dimensionality, stacking registry, and channel continuity; (ii) coordination, characterized by the identity, density, and spatial arrangement of lithiophilic sites and framework charge on pore walls; (iii) coherence, characterized by crystallinity, domain size, and channel alignment that determine whether molecular‐scale corridors remain continuous across the membrane thickness; and (iv) hybridization, where mobile phases (e.g., tethered polymers or confined plasticizers) are incorporated to tune transport behaviour. Throughout this manuscript, we adopt a cause‐effect‐performance perspective: each design lever is discussed in terms of the structural origin, the resulting changes in Li^+^ speciation and migration energetics (barrier distribution, anisotropy, and pathway continuity), and the performance signatures captured by ionic conductivity (σ), activation energy (E_a_), lithium‐ion transference number (t_Li+_), and effective conductivity in practical membranes (σ_eff_) [23]. Despite these possibilities, translation to practical solid‐state electrolyte membranes is limited by non‐idealities, namely linkage‐dependent moisture tolerance (especially for boronate ester and some imine frameworks), stacking disorder and finite crystalline domains, poor densification that increases intergranular resistance, and the inability of many COFs to form defect‐free, free‐standing films without hybrid processing. These practical limitations motivate the microstructure‐aware and hybrid design strategies highlighted in Sections 4, 5, 6, 7.

By systematically investigating the influence of building‐block geometry, linkage chemistry, framework charge, and pore‐wall functionality on ion‐highway connectivity, coordination, and coherence, this review aims to provide a mechanistic blueprint for rational COF electrolyte engineering. We critically compare representative COF architectures using quantitative transport metrics and highlight cases in which microstructure and membrane processing control σ_eff_, even when intrinsic channel transport is favourable. One conceptual distinction underlies the discussion throughout and merits early clarification: the conductivity measured with an ideal, well‐ordered channel (σ) sets the theoretical upper bound on COFs capability, while a functioning device is instead governed by the effective conductivity actually realized in the fabricated device is instead governed by the effective conductivity actually realized in the fabricated membrane (σ_eff_), The gap separating these two quantities is populated by grain boundaries, misaligned domains, residual porosity, and suboptimal electrode–membrane interfaces‐any of which can degrade transport performance by several orders of magnitude. Rapid intrinsic channel transport is thus a necessary but insufficient condition for achieving strong device‐level outcomes. This work aims to help move the field beyond empirical trial‐and‐error and toward a predictive, device‐informed framework for engineering ion‐conductive pathways in solid‐state lithium batteries.

## 2 Fundamentals of COFs for Electrolyte Applications

COFs represent a revolutionary class of organic materials that are chemically assembled from discrete molecular building blocks into two‐ or three‐dimensional (2D or 3D) extended frameworks interconnected by strong covalent bonds [24]. In contrast to conventional polymers, which are typically characterized by amorphous, disordered entanglement, COFs possess a defining feature: long‐range structural periodicity and permanent, ordered porosity [25]. This inherent crystallographic order is achieved through reversible bond‐forming reactions, collectively known as dynamic covalent chemistry (DCC). These reversible processes provide an intrinsic error‐correction or “proof‐reading” mechanism during framework assembly, enabling the formation of thermodynamically favoured and structurally well‐defined architectures [26, 27]. The modular nature of COF synthesis further permits precise control over key framework characteristics, including pore size and shape, network topology, and the identity and spatial distribution of functional groups lining the channel walls [17].

The distinctive structural features of COFs provide significant advantages when repurposed from traditional applications (such as gas storage, separation, and catalysis) [28] for use as a key component in SSEs in LIBs. Fundamentally, COFs are composed exclusively of light elements (C, H, O, N, B), which gives them an intrinsically low density, a desirable characteristic for maximizing the gravimetric energy density of next‐generation batteries [29]. More critically, the covalent backbones can provide high thermal stability; however, chemical/hydrolytic stability depends on the linkage chemistry (e.g., boronate ester and imine linkages can be moisture‐sensitive). β‐keto‐enamine and other stabilized linkages are typically more robust, often maintaining structural integrity up to 400°C or higher, a safety feature that addresses the inherent flammability risks associated with liquid organic electrolytes [30, 31, 32]. In conventional solid polymer electrolytes (SPEs), ion transport is strongly coupled to the segmental motion of polymer chains. Consequently, their room‐temperature ionic conductivity is often limited, particularly at low temperatures, where increased crystallinity suppresses polymer‐chain mobility [33]. In contrast, the cross‐linked COF scaffold remains structurally rigid over a broad temperature range and does not exhibit a conventional polymeric glass‐transition temperature (*T_g_*). Instead, ion‐transport kinetics are governed primarily by the mobility of confined guest species and by local rearrangements within the Li^+^ coordination environment under nanoscale confinement [34]. Nevertheless, many COFs are obtained as brittle, microcrystalline powders that are difficult to densify into defect‐free, free‐standing membranes. Hybrid‐processing approaches, including polymer infiltration, binder‐assisted consolidation, and oriented film growth, are therefore often required to reduce intergranular resistance and produce mechanically robust, scalable electrolyte architectures [35, 36].

From an ion‐transport perspective, the distinct morphology of two‐dimensional COFs with stacked layers held together by weak π–π interactions yields highly desirable one‐dimensional (1D) nanochannels that extend throughout the material [22, 29]. This is the crucial structural difference from conventional SSEs: in inorganic solid electrolytes (ISEs), ion transport typically occurs via defect‐mediated hopping within a rigid (tortuous) lattice, whereas in COFs, the ordered nanopores provide a continuous, relatively straight pathway for ion migration [30]. This mechanism is conceptually distinct from the segmental motion observed in polymers and the defect‐dependent hopping mechanism in ceramic electrolytes, thereby establishing the COF framework as an ideal scaffold for high‐speed, directional ion transport. For reference, many 2D COF electrolytes report pore diameters in the ≈1–3 nm range and interlayer spacings of ≈3.3–3.8 Å. Reporting these values, along with the stacking registry and texture, is important for relating channel geometry to σ_eff_ [21, 36].

The fundamental concept of COF utilization in electrolytes, as broadly illustrated in previous review works, can be summarised in four strategies that may be classified according to the charge properties of the COF skeleton itself [37, 38]. The first category involves neutral COFs that act as uncharged yet chemically active hosts (often with carbonyl/ether/pyridine/imine donors) that impose structural ordering. In this role, the fixed‐pore structure of the COF exerts an important nanoconfinement effect on guest molecules, such as lithium salts, plasticizers, polymer chains (e.g., poly(ethylene glycol)), and ionic liquids [35, 39, 40]. Such confinement can inhibit the recrystallization of encapsulated polymers at room temperature, thereby reducing the interaction between Li^+^ cations and their counterions (ClO^4−^ or TFSI^−^) and promoting the dissociation of lithium salts, thereby increasing the concentration of free and mobile Li^+^ ions [30, 35]. The second and third strategies correspond to the so‐called ionic COFs (ICOFs), which are synthesized by incorporating either permanently fixed anionic moieties or cationic moieties into the backbone [34, 41]. Anionic COFs, usually containing groups such as sulfonates, carboxylates, or imidazolates, host in situ Li^+^ binding sites that function as single‐ion conductors by immobilizing the negative charge carriers, thereby achieving excellent lithium‐ion transference numbers (t_Li+_) close to unity [36, 42]. Cationic COFs, on the other hand, contain positively charged groups such as guanidinium or imidazolium and work by electrostatically coordinating and immobilizing the lithium salt anions (such as TFSI^−^); this effect is quite similar in its ability to decouple the Li^+^ cation, thereby facilitating its preferential transport [43]. A fourth emerging class is Zwitterionic or mixed‐charge COFs, in which spatially separated positive and negative moieties are incorporated into an overall electroneutral scaffold to produce internally polarised pore surfaces that promote salt dissociation while inhibiting anion mobility [44].

In essence, the underlying appeal of COFs as electrolyte components is that they offer the possibility of uniting the tunability and flexibility of organic chemistry with the long‐range order and mechanical stability traditionally associated with crystalline inorganic solids. This synergy provides a promising platform for designing electrolytes that address the main deficiencies of current state‐of‐the‐art SSE materials, i.e., the trade‐off between electrical conductivity and mechanical/thermal stability [34]. By leveraging their inherent structural properties, researchers aim to move beyond simple inclusion complexes toward the design of integrated chemical systems in which the COF skeleton actively determines the mechanism of ionic conduction. The overall conceptual framework of this review is summarized in Figure 2.

**FIGURE 2:**
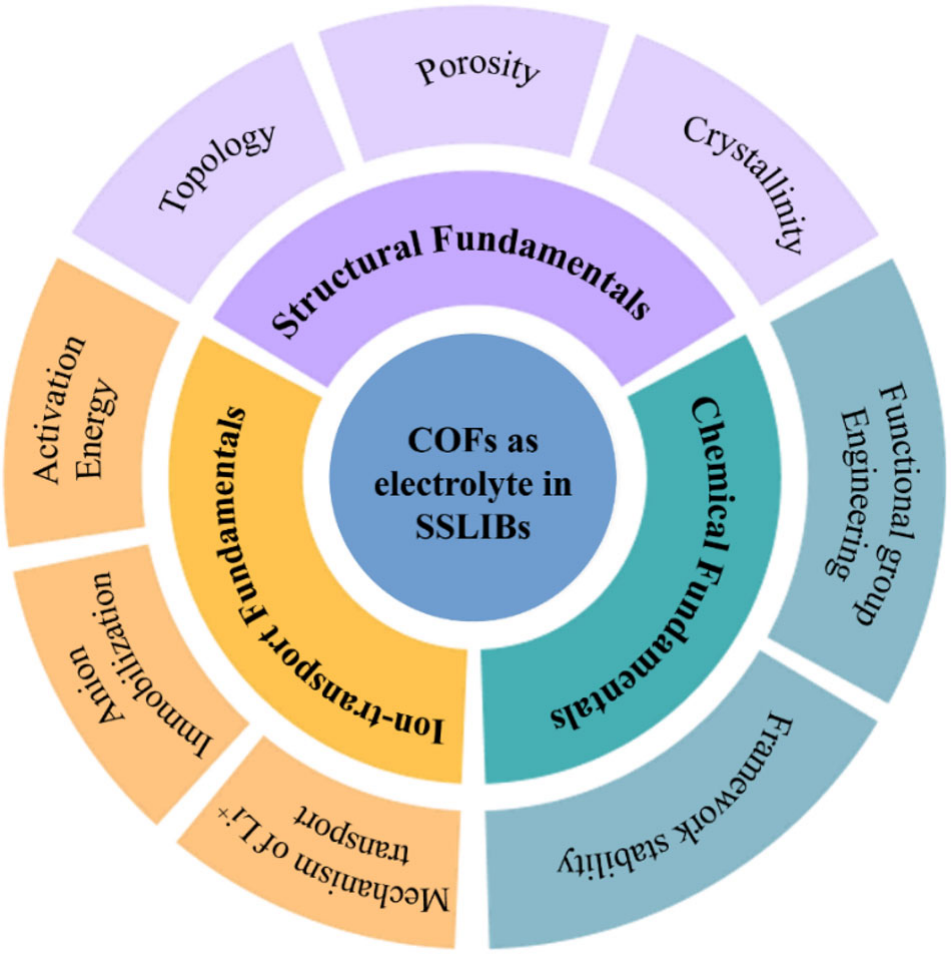
Schematic overview of covalent organic frameworks as electrolytes for solid‐state lithium batteries, highlighting their structural features, chemical characteristics, and Li^+^‐transport fundamentals.

### 2.1 Design Rules of Hybrid COF Electrolytes

Several practical design rules can be derived from the emerging literature on COF‐based hybrid electrolytes. First, the hybrid microstructure should be engineered to ensure percolation; regardless of whether the COF serves as the primary transport pathway, the polymer or confined phase must span particle‐contact interfaces and eliminate transport‐inactive voids. Second, the pore‐wall chemistry of the COF should be selected with consideration of the target salt‐carbonyl, pyridinium, sulfonate/sulfonimide, or carboxylate motifs, which are known to shift the dissociation equilibrium and strongly affect enhanced transference behavior [45, 46, 47, 48]. Third, tether density and pore size must be co‐optimized in polymer‐in‐framework systems: excessive crowding can increase activation barriers despite increasing the density of solvating sites, whereas insufficient tethering fails to establish a continuous solvating landscape [49, 50].

Finally, studies on hybrid COF electrolyte would benefit from standardized disclosure of (i) ionic conductivity over a wide temperature range with activation energies, (ii) Li^+^ transference numbers measured under comparable conditions, (iii) mechanical properties pertinent to the membrane handling, and (iv) compositional descriptors that enable meaningful comparisons (COF loading by wt% and/or vol%, pore‐filling fraction, and salt‐to‐ether or salt‐to‐functional‐group ratios). Such reporting will be critical to advancing the concepts of hybrid design toward reproducible, scalable electrolyte formulations required for next‐generation solid‐state lithium battery design [45, 46, 47, 49, 51]. The descriptor‐to‐metric perspective is summarized schematically in Figure 3.

**FIGURE 3:**
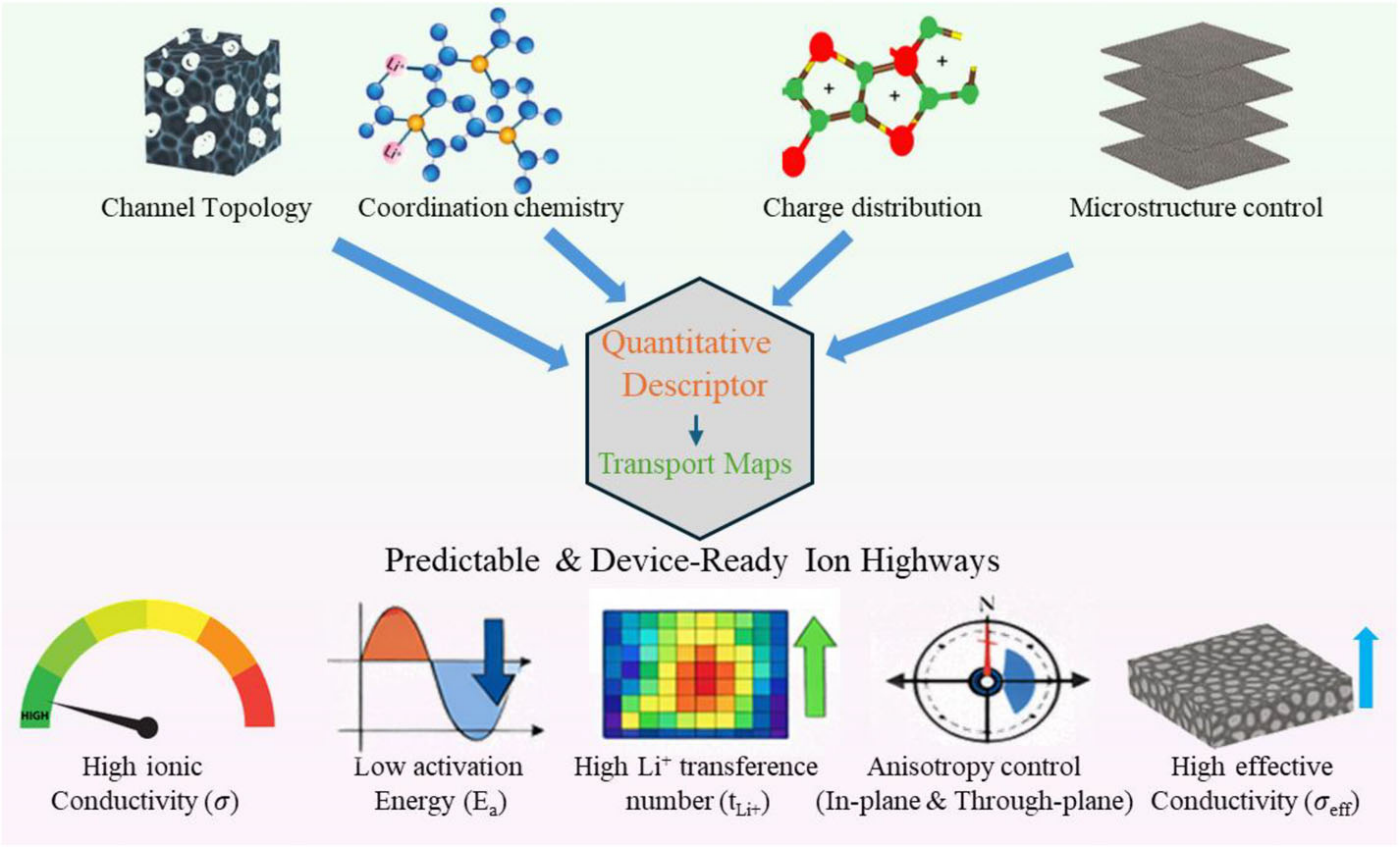
Schematic of descriptor‐driven ion transport in COF electrolytes linking structure to key metrics (σ, Ea, t_Li+_, anisotropy, and σ_eff_).

### 2.2 Why Design Rules Are Still Missing: Need for a Descriptor to Metric Maps

Despite the demonstrable success of certain COF‐based electrolyte systems, the design of Ion Highway is still largely empirical. Many high‐performing materials have been discovered through targeted synthesis and trial‐and‐error optimization, rather than through universally applicable and quantitative rules [52]. Although it is well known that pore diameter, stacking arrangement, functional group identity/density, and alignment have a strong influence on transport, a predictive framework that relates molecular level descriptors (e.g., pore size distribution, donor density, stacking registry, crystallinity/domain size) to macroscopic transport parameters [σ, E_a_, t_Li+_, anisotropy, σ_eff_] across a broad range of COF families is still lacking.

Individual case studies emphasize why generalization remains challenging. For example, the conductivity of imidazolate COFs with electron‐withdrawing substituents (e.g., CF_3_) can be enhanced by negative charge delocalization and reduced Li^+^ association [52]. In contrast, in other systems, increasing pore size may dilute effective coordination sites or charge carriers within a channel, resulting in lower conductivity even though a nominally wider channel is formed [53]. Likewise, the influence of stacking modes (AA vs. slipped AA′ or AB) on axial versus planar ion conduction has not been conclusively resolved in all studies, partly because many reports involve composite or multi‐component electrolytes in which the respective host and guest species are not fully deconvoluted [33, 54].

## 3 COFs as Ion Highways

The concept of “Ion Highways” summarises the key hypothesis of the research program developing the COF electrolytes: the intrinsically ordered, crystalline nanochannels of COFs—often quasi‐one‐dimensional (1D) along the pore axis—can be engineered to create low‐resistance, directional pathways for Li^+^ transport in solid‐state systems [32]. In contrast to ion conduction in highly disordered solids (where migration can be dominated by heterogeneous hopping across random sites) or in conventional polymers (where transport is coupled to sluggish segmental relaxation), COF channels offer a structural route toward geometry‐defined Li^+^ migration with the possibility of controllable transport anisotropy [32]. In this view, the COF framework is not simply a mechanical scaffold but a transport architecture, in which pore geometry, wall chemistry, and long‐range order together determine the thermodynamic and kinetic landscape experienced by mobile ions.

### 3.1 Ion Transport Within COF Channels: Hopping Versus Vehicular and Anisotropic Migration

Ion transport in COFs is often modelled by two modes, which are limiting: (i) site‐to‐site hopping between coordination sites on the pore wall surface, and (ii) vehicular transport in which Li^+^ migrates as part of a solvated complex or polymer/ionic liquid‐assisted environment inside the pore volume. The dominant regime is dependent on pore filling, the strength and periodicity of Li^+^‐framework interactions, and the local solvation structure established under confinement [36]. A defining mechanistic signature of the Ion Highway concept is anisotropic conduction: Migration along the channel axis is often more favorable than transport across layers or between neighboring pores. Density functional theory (DFT) calculations of axial and planar migration barriers frequently show a reduction in the axial barrier, consistent with the idea that the ordered, periodically functionalized channels facilitate migration along this direction [55]. For example, β‐ketoenamine TpPa‐SO_3_Li has been reported to exhibit a substantially lower barrier for axial migration (∼0.18 eV) than for planar transport, indicating that internal geometry and coordination‐site organization can create a preferred conduction pathway at the atomic scale [41, 56]. Collectively, these mechanistic insights support a key inference: Ion Highways are generated when confinement and periodic coordination lower the activation barriers for axial migration while preserving pathway connectivity. Importantly, barriers and diffusion trends calculated for idealized pore models do reflect intrinsic mechanistic tendencies but do not directly predict σ or E_a_ in real membranes, where microstructural factors (pellet density, grain boundaries, tortuosity) and pore occupancy may dominate the observed transport response [22, 36].

### 3.2 Design Levers for Ion Highways: Pore Size, Pore‐Wall Chemistry, and Alignment

Realizing COF‐based ion highways requires the integrated optimization of four interdependent design elements: (i) pore size and channel connectivity, (ii) pore wall chemistry (functionalization and framework charge), (iii) crystallographic order and alignment (topology/microstructure coherence), and (iv) hybridization strategies (introducing mobile conducting media within otherwise rigid channels when insufficient mobility at room temperature is insufficient). The nanochannel environment imposes confinement that can modify solvation and migration barriers compared with bulk electrolytes. In particular, by matching the pore diameter with the effective size of Li^+^ solvation, the frequency of productive Li^+^‐wall interaction can be increased, thereby facilitating rapid hopping along the channel axis [36].

A second design lever involves periodically functionalizing the pore walls. When Li^+^‐coordinating groups are fixed at regular intervals, a sequence of continuous “handoff” sites is created, which reduces the effective activation barrier for axial migration. COFs functionalized with imidazolate [57] and sulfonate‐based motifs [56] exemplify this concept: the pore walls act as programmable coordination landscapes that can support repetitive Li^+^ coordination–decoordination events during migration. Importantly, the magnitude of Li^+^ binding has to be balanced—it may be too weak (leading to reduced stabilisation/dissociation of the carrier) or too strong (leading to trapping of Li^+^ and increased Ea, which means that donor identity and density are important parameters for rational optimization [22, 55]. These key ion‐highway design elements are summarized schematically in Figure 4.

**FIGURE 4:**
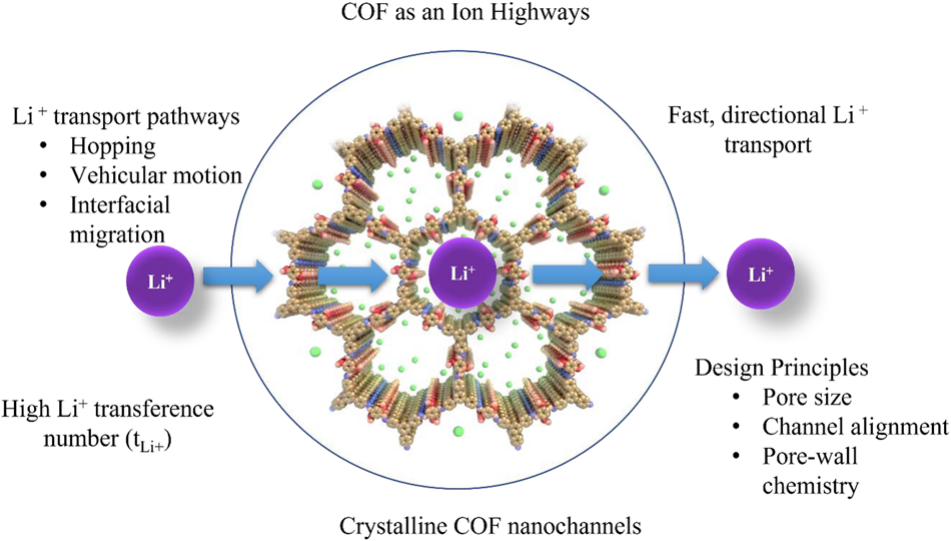
COF nanochannels act as ion highways in solid‐state LIBs, enabling fast directional Li^+^ transport via optimized pore size, alignment, and chemistry.

The third design lever concerns crystallographic order and alignment, which control the continuity of transport pathways and the extent to which directional transport can be realized at the device level. Variations in stacking (AA vs. slipped AA′ or AB) and the extent of texture/alignment can affect the axial vs. planar transport, thereby determining whether the intrinsic channel advantage is translated into effective conductivity and transport anisotropy in membranes/pellets [56].

### 3.3 Host–Guest Synergy: Confined Polymers/ILs and In Situ Polymerized Electrolytes

Beyond single‐component pore filling, Ion Highways increasingly rely on host–guest architectures, in which the COF framework and an incorporated electrolyte phase synergistically enhance ion transport. A common approach is to trap low‐molecular‐weight polymers (e.g., PEO) or lithiophilic ionic liquids (ILs) within COF channels [33, 43]. Under confinement, the rigid pore walls can inhibit polymer crystallization and maintain a more amorphous, liquid‐like segmental environment even at near‐room‐temperature conditions, thereby enhancing Li^+^ mobility compared to bulk polymer electrolytes [49].

More recently, “electrolyte mediation” approaches have been reported, wherein the basic structure of a polymer electrolyte (e.g., dimethylacrylamide, DMA) is introduced in the form of a polymerizable liquid, which is infused into the pores of the COF and polymerized in situ to create a confined, percolating phase for ion transport and supported by the ordered channel geometry. Such confined polymerization can simultaneously enhance pathway continuity and Li^+^ transport performance, achieving some quite impressive room‐temperature values (σ ≈ 1.65 × 10^−4^ S cm^−1^; t_Li+_ = 0.85) in representative systems [54, 58]. These results highlight a more general design rule: The most successful Ion Highways often arise when the pore architecture (structure) and pore occupancy (guest phase) are designed in concert, rather than treating one as given and optimizing the other empirically.

## 4 Structure Activity Relations With Performance Metrics of COFs

Compared with ceramic SSEs (conductance dependent on defect chemistry, which is not trivially programmable) and polymer electrolytes (where transport is coupled to segmental dynamics), COFs allow modular “molecular coding” of corridor geometry, donor fields, and fixed charges, thereby providing a more direct descriptor to mechanism to metric mapping. The ion‐highway design framework used throughout this section is summarized in Table 1.

**TABLE 1:** Ion‐highway design framework linking COF descriptors to transport effects and performance signatures.

Design lever	Controllable descriptor (cause)	Transport‐level effect	Performance signature
Connectivity	Topology, channel dimensionality, stacking registry, channel orientation	Pathway continuity and tortuosity; anisotropy of migration corridors	σ, E_a_, anisotropy, σ_eff_
Coordination	Donor identity/density; fixed framework charge; spatial arrangement of sites	Li^+^ speciation, residence time, hopping frequency, barrier landscape smoothness	σ, E_a_, t_Li_^+^; carrier fraction
Coherence	Crystallinity, domain size, misorientation, and grain‐boundary density	Continuous long‐range corridors; reduced bottlenecks between crystallites	σ_eff_/σ gap; temperature dependence
Hybridization	Confined/tethered polymer or quasi‐solid media inside corridors; guest loading	Added segmental dynamics and solvation within an ordered corridor	Room‐temperature σ and σ_eff_ at reduced thickness

### 4.1 Topology

Topology in COF‐based solid‐state electrolytes can be most appropriately interpreted as a hierarchy of geometrical constraints that collectively regulate ion transport across multiple length scales: (i) the crystallographic net (2D vs. 3D connectivity), (ii) the pore structure (cross‐section, aperture distribution, and internal void volume), (iii) the stacking registry and interlayer coherence that lead to the merging of pores into continuous axial tunnels, and (iv) the mesoscale assembly of crystallites into dense films or composite membranes, which ultimately defines percolation and tortuosity. As solid‐state conductivity and critical current density are system properties, the same chemical functionality can provide very different electrochemical results, depending on whether topological continuity is maintained from the unit cell to the electrode–electrolyte contact region.

Within a structure–activity relation framework, topology therefore operates as a geometric “operating system” that determines (a) whether Li^+^ encounters a continuous path of lowest energy or a sequence of dead‐ends, (b) whether counter‐anions can be immobilized without disrupting percolation, and (c) whether the intrinsic anisotropy of layered COFs can be translated into device‐scale transport. Importantly, topology is not limited to idealized nets, and practical electrolytes contain defects, stacking disorder, grain boundaries, and polymer‐COF contact regions, where all of these can play a dominant role in the effective transport length and interfacial resistance

#### 4.1.1 Network Topology as the Main Descriptor of the Dimension of the Channel

COFs are constructed from organic building blocks of known symmetry (linear, trigonal, tetragonal, tetrahedral) into periodic network topologies. In terms of electrolyte function, the most direct effect of the net topology is the dimensionality and directionality of the resulting void network. 2D layered nets generally produce 1D channel pathways when stacked together, but many 3D nets from multidirectional pathways are less sensitive to crystallographic misorientation.

The archetypal layered‐channel topology is represented by COF‐5, a stacked 2D framework that forms honeycomb‐like pores with diameters approaching 30 Å and generates 1D tunnels perpendicular to the layers. As shown in Figure 5, the channel structure of COF‐5 and the Li^+^ diffusion pathway illustrate 1D transport along the pore axis. Ab initio molecular dynamics simulations for a model electrolyte consisting of a carbon monoxide functionalized graphene material (COF‐5)/LiClO_4_/THF electrolyte demonstrate that diffusion of Li^+^ ions is mostly restricted in the tunnel interior region and may exhibit 1D transport behavior with liquid‐like behavior (coordinated rearrangements of solvent molecules and anions within the channel) [59].

**FIGURE 5:**
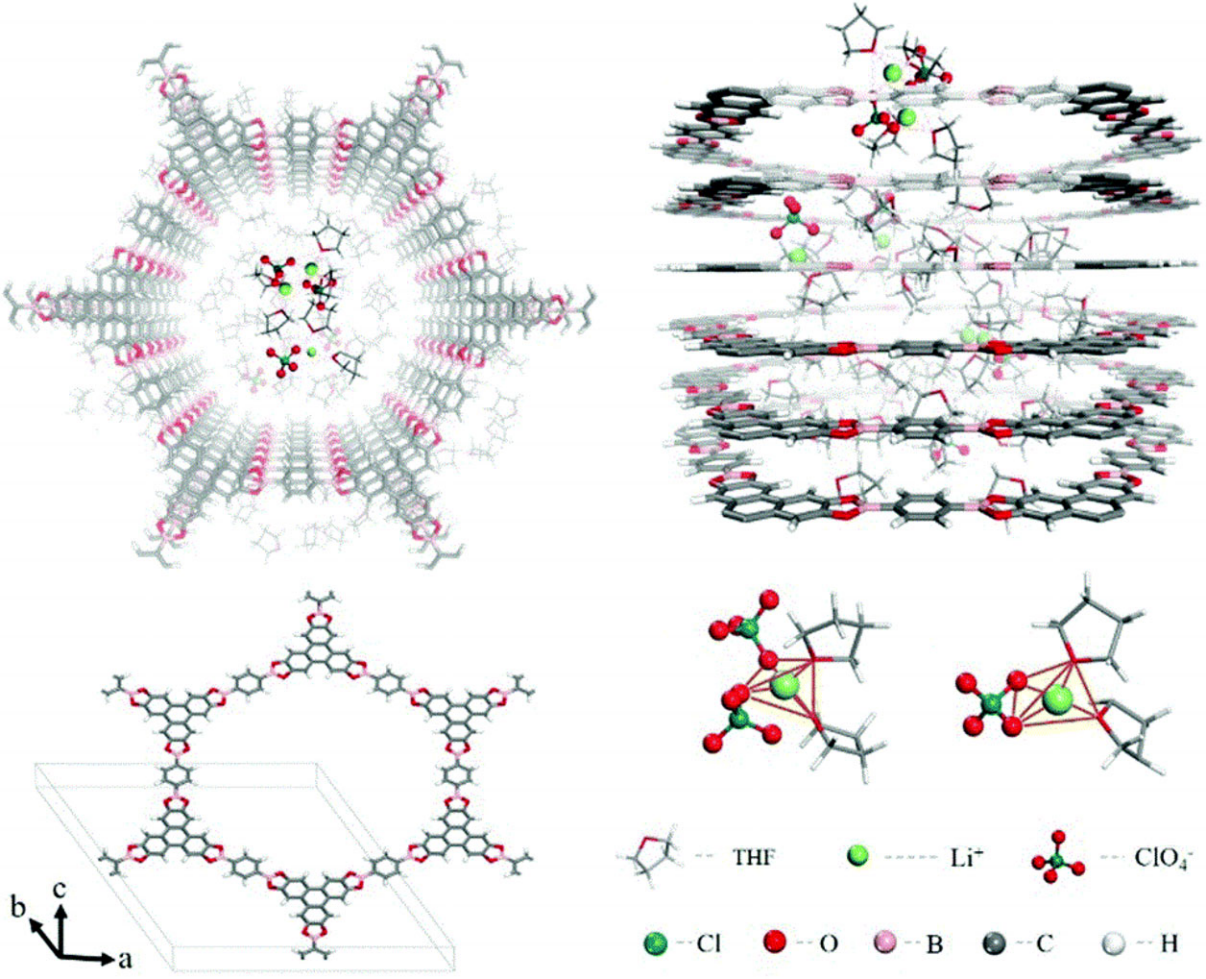
Channel structure of COF‐5 and Li^+^ diffusion pathway, illustrating one‐dimensional transport along the pore axis. Reproduced with permission [59].

However, COF‐5 additionally demonstrates an important drawback of “large‐pore” channel topology, and indeed the broad tunnel does not necessarily ensure high conductivity if the microenvironment of the pores does not sustain a high density of mobile carriers and a low coordination–rearrangement barrier. In confined electrolytes, there is a risk of excess pore volume, in which charge carriers are diluted, thereby reducing ion–ion interactions with ions/solvents necessary to sustain concerted migration and increasing sensitivity to stacking imperfections, leading to disruption of axial continuity. Beyond archetypal boronate COFs, most electrolyte‐oriented 2D COFs adopt a hexagonal or related motif, generating long 1D channels when eclipsed (or near‐eclipsed) stacking occurs, thereby bringing the “ion highway” concept to life. Thiophene‐based imine COFs (COF‐NUST series) are an instructive example: structure optimization and refinement based on a bnn net result in a predicted crystallization within the P3 space group with ordered nanochannels, whereas gas sorption is used to reveal microporosity with characteristic pore sizes of about 20.4, 21.2, and 12.4 Å for different isoreticular members. Although these materials are frequently exemplified by channel‐confined ionic liquid/Li salt phases, the salient point is that the net topology dictates a channel direction and an accessible cross‐section, which can be varied systematically by linker choice [33].

A parallel design direction utilizes 3D COF net topologies to combine ordered channels with improved mechanical robustness. In imine‐linked 3D porous COFs based on the diamond topology, the framework has been shown to generate 1D rectangular channels along the crystallographic *c*‐axis, providing a topological pathway for oriented transport within a mechanically continuous 3D scaffold [60]. In highly conjugated 3D COFs, sevenfold‐interpenetrated diamond nets have been reported (dia‐c7), where interpenetration increases material density, improving mechanical integrity while retaining permanent porosity and open void space for ion hosting [61].

Overall, net topology regulates not only the presence of 1D channels but also the extent of intrinsically anisotropic transport. Layered 2D nets can provide exceptionally low axial tortuosity, provided that stacking is well controlled. In contrast, 3D nets can reduce dependence on macroscopic orientation and the resulting performance penalty associated with randomly oriented crystallites in pelletized electrolytes [60, 61]. Thus, topology selection (2D vs. 3D; degree of interpenetration) is a practical lever for controlling transport continuity during processing: 2D nets' advantages include texture/registry control and the ability to enable through‐thickness percolation, while 3D nets provide multidirectional connectivity and greater tolerance to misorientation. However, they remain sensitive to densification and interparticle contacts.

#### 4.1.2 Pore Topology: Cross‐Section, Aperture Distribution and Confinement‐Driven Selectivity

Beyond the abstract network description, pore topology describes the locally experienced geometry of ions: pore cross‐section (hexagonal vs. rectangular), aperture distribution (single mode vs. hierarchical), and the internal free volume available to solvates, polymer segments, and counterions. A recurring observation across COF electrolytes has been that larger pores are not necessarily superior. Rather, there is an optimum confinement regime to meet (i) the need for space for coordination rearrangement and segmental motion (if polymers are present) and (ii) the need for low enough interactions with pore‐wall functional groups to ensure high carrier density and ion selectivity. Accordingly, no single pore‐size window consistently maximizes Li^+^ transport in any given COF. The optimized pore diameter must be system‐specific and co‐optimized with the pore‐wall chemistry and pore occupancy (salt loading, plasticizer/polymer content) to avoid either a carrier‐dilution phenomenon (excessively large pores) or steric/kinetic bottlenecks (excessively small and/or crowded apertures). In this sense, topology is primarily responsible for determining the geometric component of selectivity (confinement, tortuosity, aperture statistics). At the same time, the pore wall chemistry determines the energetic components (donor strength and fixed charge), and the observed t_Li+_ results from their interplay.

Electrolyte‐mediated COF systems provide a representative case study of how pore topology can serve as a microenvironment for engineering effective ion transport. In such a case, for example in a lithiated sulfonate COF (LiCOF) host, the dominating intrinsic channel size is around 1.8 nm, and through the phase loading of polymerizable electrolyte (i.e., DMA@LiTFSI), the accessible pore space of the COF is partly occupied or restructured and the “working” pore topology experienced by Li^+^ is effectively altered to decrease the void‐driven contact resistance at grain boundaries. In these systems, pore topology is inextricably linked to the mesoscale topology formed by pore filling and interparticle “welding” during electrolyte mediation [53].

Pore topology is also of key importance in COFs designed as anion‐immobilized single‐ion conductors. A representative anion‐immobilized keto‐enamine COF assembled as a hexagonal unit is described, having a pore size of ∼1.63 nm and an interlayer distance of ∼0.34 nm, which is an important observation showing how well‐defined pore diameter and stacking period can be maintained even in the case where bulky anionic moieties (e.g., TFSI‐derived groups) are incorporated into the backbone. In this case, pore topology has to take into account both fixed anions and the mobile Li^+^, such that the available cross section and the spatial periodicity of the charges along the pore channel make the conductivity and the transference behaviour co‐determinants [62].

Zwitterionic pore‐wall functionalization is an alternative approach to modifying pore topology and internal electrostatics. In a pyridine‐rich parent COF (NCOF), post‐functionalization to produce a zwitterionic COF (Zwitt‐COF) preserves stacked porous structure but alters the internal charge distribution and the effective pore atmosphere; the reported room‐temperature ionic conductivity is 1.65 × 10^−4^ S cm^−1^, which represents how well‐designed the pore microenvironments can support fast transport without liquid‐electrolyte infiltration. The important topological conclusion is that the same net may exhibit different “functional” pore topology when the accessible free volume is reshaped, and the association of ions within the channel is regulated [63].

Flexible side‐chain engineering extends into the pore‐topology design space by inserting “soft” internal boundaries that regulate both the pore diameter and local solvation. In electrospun composite organic membranes based on PEG‐functionalized COFs, systematic variation of side‐chain length allows tuning of pore size from ∼2.38 to ∼1.89 nm, with a significant decrease in BET surface area from ∼242.9 to ∼21.4 m^2^ g^−1^, corresponding to the continuous occupation of the internal void volume by tethered chains [35]. This reflects a topology‐chemistry coupling, in which the framework net remains periodic; however, the effective cross‐section of the pores and the internal free volume are redefined by side‐chain packing, thereby enabling control of the carrier concentration and coordination dynamics.

Vinylene‐linked COFs provide a complementary example in which pore topology is combined with better chemical stability. A vinylene‐linked 2D COF (VCOF‐1) has been reported with AA stacking and cylindrical pores; according to gas sorption measurements, the surface area is high (∼1364 m^2^ g^−1^), and the pore size distribution is centered at ∼1.50 nm. When such a framework is incorporated as a low‐loading additive into polymer electrolytes, the cylindrical pore topology can serve as a directional channel for Li^+^ transport, with the polymer host facilitating it. At the same time, the nonpolar vinylene linkage favors a stable framework‐Li metal compatibility [64].

At the opposite end of the pore size range, comparably large‐pore COFs have often been employed as additives in polymer electrolytes rather than as single‐channel conductors. A benzothiadiazole/triazine COF (CPs‐BT) introduced at a low loading into PEO‐based composites has a BET surface area of ∼67.81 m^2^ g^−1^ and an average adsorption pore diameter of ∼6.85 nm, suggesting a mesoporous topology, which can lower polymer crystallinity and occasionally provide fast transport shortcuts at which the polymer segmental motion alone would be limiting. In such additive‐enabled composites, the pore topology plays a less dominant role in the structure by enforcing strict 1D confinement of the pore geometry and a more dominant role in filling available void space and in polymer‐COF contact regions, which are responsible for the redistribution of salt and the suppression of crystallinity [65]. Representative COF‐solid‐state electrolyte systems, along with their reported pore sizes and specific surface areas, are summarized in Table 2.

**TABLE 2:** Representative COF‐based solid‐state electrolyte systems, summarizing pore size, specific surface area, and corresponding references.

COFs (system)	Pore size (nm)	Surface area (m^2^ g^−1^)	Reference
LiCOF to DLC (DMA@LiTFSI mediated COF electrolyte)	1.8 (dominant COF channel)	508	[53]
EB‐COF‐TFSI additive in PEO SSE (1%EB‐COF)	—	748.7	[66]
COF‐MCMC (gate‐functionalized COF SSE)	—	829.0	[25]
Imidazolate COF with ether chains (Li^+^@PI‐TMEFB‐COFs SSE)	4.9 (average pore diameter)	80	[67]
VCOF‐1 filler in PEO SPE (VCOF‐SPE)	1.50 (pore size distribution center)	1364	[64]
COF‐ECJTU1‐X defective COF series	1.27 (pore peak)	810–570 (X = 0–60)	[68]

#### 4.1.3 Stacking Topology: Registry, Slippage, and Continuity of Ion Highways

For layered 2D COFs, the stacking topology is often the most influential topological variable because it determines whether pores form continuous, low‐tortuosity axial tunnels or offset, kinked, and partly blocked ones. The ionic conductivity measured in pellets or films is thus very sensitive to control over registry (AA/AB/AA′‐type stacking), interlayer spacing, and the spatial coherence length of aligned channels leaving the crystallites. In practice, stacking/registry effects frequently control σ_eff_ through device‐scale percolation and boundary crossings. In contrast, the impact of salt dissociation and t_Li+_ is more directly controlled by the pore wall chemistry. Decoupling the two contributions requires topology‐controlled series with constant chemistry, and vice versa.

A structurally resolved illustration is given by the imine‐linked COF (I‐COF) and cyclization‐modified derivative (Q‐COF). Structural modeling suggests that the parent I‐COF can adopt an AA′‐2.6 stacking mode, with characteristic reflections consistent with ordered layers. In contrast, post‐synthetic Povarov‐type cyclization forms a Q‐COF with a modified linkage that retains a layered framework while enhancing interlayer molecular interactions and electrochemical robustness. As illustrated in Figure 6a, this stacking topology is directly connected to transport: Q‐COF has uniformly oriented channels during processing, enabling a continuous axial “ion highway” that supports stable Li plating/stripping and high‐voltage operation with nickel‐rich cathodes. Stacking effects are also present in chemically functionalized systems. In Zwitt‐COF, TEM analysis shows that the stacked structure is preserved upon zwitterion formation. Still, the decrease in crystallinity and the change in stacking preference observed in Figure 6b,c, together with theoretical models emphasizing AB‐type stacking motifs, indicate that pore continuity and interlayer coherence can still be altered even if the main net remains unchanged. In practice, this means that “identical” pore diameters extracted from building blocks may produce different conductivities when the stacking registry and disorder are modified by post‐functionalization.

**FIGURE 6:**
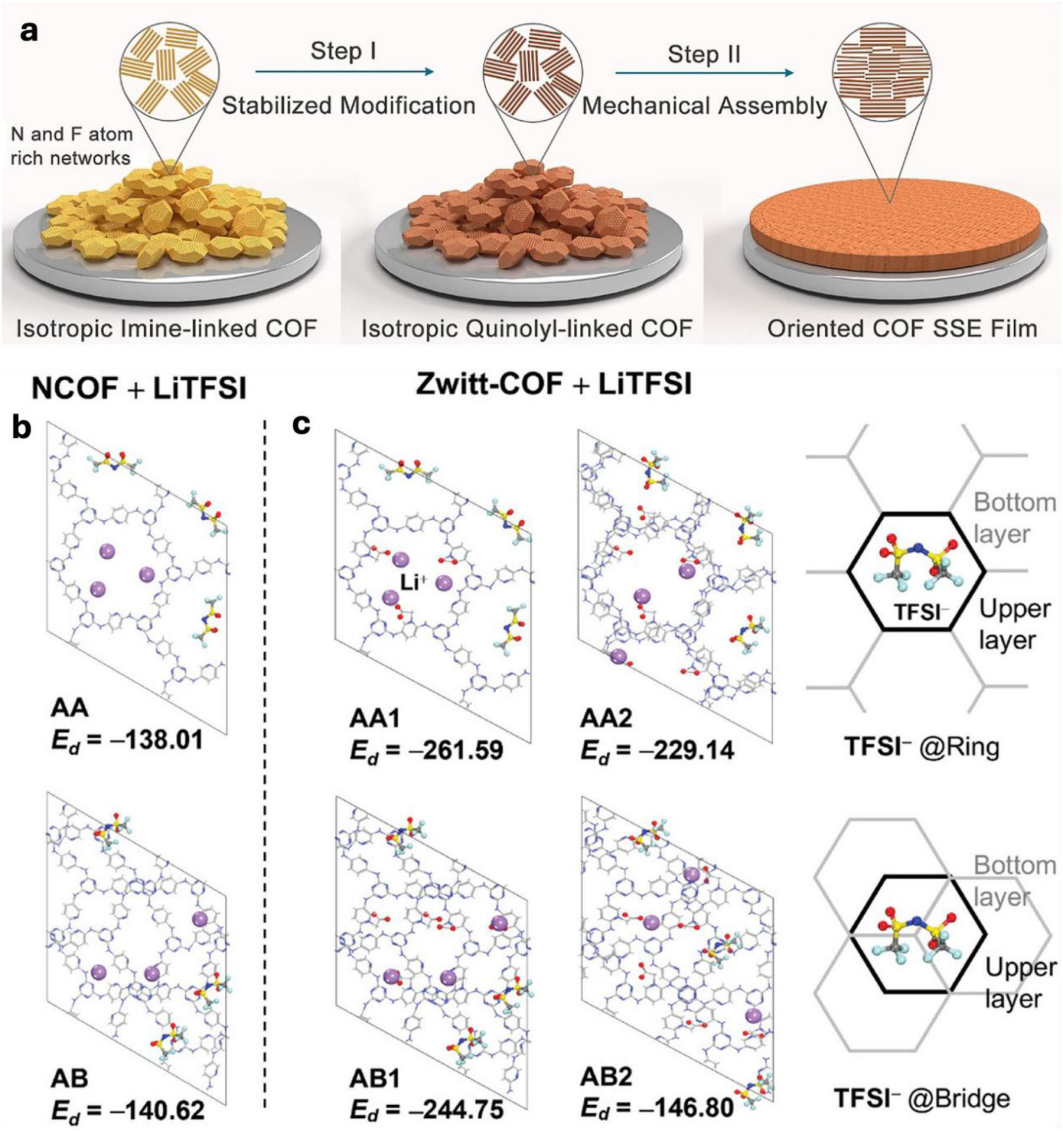
(a)Two‐step fabrication scheme for a COF electrolyte film with holistically oriented ion‐transport channels for through‐thickness Li^+^ conduction. Reproduced with permission [22] (b, c) stacking configurations and Li^+^ dissociative adsorption in Zwitt‐COF, highlighting structural reconstruction toward AA stacking and the formation of linear hexagonal ion channels. Reproduced with permission.

In polymer‐COF composite electrolytes, stacking topology remains relevant, as polymer infiltration and salt coordination can stabilize or destabilize certain registries. For VCOF‐1, the registry of PXRD matches between experiments and simulations reveals a highly ordered pattern, which is favorable to the cylindrical channel axis for directional Li^+^ migration when embedded in PEO‐based matrices. Similarly, PEG‐functionalized COFs within electrospun membranes maintain layered order but modify effective pore apertures through side‐chain packing, so that the stacking and pore topologies are connected and therefore co‐engineered, rather than separate processes.

#### 4.1.4 Crystallographic Orientation and Mesoscale Topology: The Translation of Channels Into Membranes

Even when the topology of the class of unit cells is favorable, macroscopic ion transport depends on the fast‐conducting axes being oriented across the electrolyte thickness and on grain boundaries that are densified, with low void content and low resistance at the interparticle level. Mesoscale topology, therefore, comprises crystallographic texture, particle packing, and the continuity of intergranular contact networks. Across reported COF electrolytes, introduction of texture/alignment and densification generally reduces tortuosity and increases σ_eff,_ though it is system‐dependent and is limited when transport is determined by grain‐boundary resistance or by pore filling and breaking the continuity of the axial direction; thus, orientation should be reported with porosity and contact impedance to uncouple its contribution.

Oriented COF films provide direct proof of the benefits of mesoscale topology control. In Q‐COF solid electrolyte thin films processed by uniaxial pressing at high temperature, a strong (001)‐type texture (as evidenced by concentrated intensity distributions in 2D diffraction) results in a densely stacked microstructure and an ultrathin thickness of ∼50 µm. The high degree of orientation allows the transport to utilize the axial channel direction across the film thickness, rather than being limited by lateral grain boundaries, thereby linking the topic‐intrinsic channel to device‐scale performance.

Electrospinning‐based fabrication of a composite organic membrane is another approach to mesoscale topology control, incorporating COF domains into a continuous network of polymer fibers and then performing density compaction. In PEG‐COM systems, we can achieve PEG2‐COM membranes as low as ∼22 µm thick (and PEG1‐COM ∼10 µm) using hot pressing, which shows the power of topology control (expressing the topology control as a decrease in macroscopic transport length position while maintaining the axial function of the channels within embedded COF domains). Because thickness reduction directly affects the ohmic contribution to polarisation, the ultrathin membrane topology can serve as a practical means to increase the volumetric energy density in full cells.

Electrolyte mediation offers a particularly effective form of mesoscale topological re‐engineering, since it simultaneously alters the microenvironment of the channels and the network at the grain boundary. In LiCOF‐derived electrolytes under electrolyte conditions, loading and in situ polymerization of DMA@LiTFSI form a deformable “liquid‐like” sub‐assembly inside a solid COF scaffold, which fills voids and decreases interparticle contact resistance and yet maintains the intrinsic topological structure of the underlying 1D channel framework; the practical topological result is an in situ welded network with lower tortuosity at the pellet scale.

Ionic COF‐framed composite architectures also exemplify mesoscale topology. An ionic liquid polymer phase is incorporated into iCOF/PIL composites to fill voids between iCOF crystallites, thereby converting an initially discontinuous packed‐bed structure into a more continuous ion‐conducting matrix without compromising the pore‐mediated ion selectivity of iCOFs. In this case, the mesoscale topology (filling voids and interparticle connectivity) has been designed to directly convert intrinsic pore/channel characteristics to low‐resistance macroscopic transport [26].

#### 4.1.5 Topology in Ionic and Charged COFs: Fixed‐Charge Distributions and Channel Electrostatics

Topology cannot be separated from electrostatics when the COF backbone is ionic, because fixed charges are incorporated into a periodic lattice. Spatial periodicity, density, and chemical softness of fixed‐charge groups at pore walls define whether Li^+^ can percolate and counter‐ions are immobilized or whether migration occurs via hopping between discrete coordination sites or through quasi‐continuous solvation channels.

Fixed‐charge topology: Anionic COFs exhibit fixed charge. The hexagonal pore shape (pore size ∼1.63 nm) and stacked‐channel structure (interlayer distance ∼0.34 nm) in anion‐immobilized keto‐enamine COFs with TFSI‐derived moieties create a periodic array of anionic sites along the channel direction. It offers a fixed‐charge topology, which may increase Li^+^ transference by preventing anion movement, though it reduces available free volume; thus, pore diameter and charge density should be co‐optimized to avoid blocking percolation without excessive anion immobilization.

Cationic COFs enhance a complementary mechanism of charge topology in polymer composite electrolytes. An additive known as cationic COF (EB‐COF) in PEO‐based solid electrolytes has a high internal surface area (BET ∼748.7 m^2^ g^−1^) and a pore volume (= 0.193 cm^3^ g^−1^), which is measured and shows a porous topology that can create a large contact area between the polymer and cationic COF and enables the accommodation of dispersed counter‐anions in the ordered void space. Such systems, charge topology, and pore topology co‐regulate salt dissociation and the spatial distribution of anions, thereby affecting the conductivity and stability of the contact [66].

A hierarchical charge topology is incorporated into Ionic COF composites. The iCOF pores in iCOF/PIL composites carry an anionic moiety that engages in competitive coordination with Li^+^ and TFSI^−^, enabling rapid Li^+^ transfer while limiting anion flux. The reported composite systems allow ionic conductivities to reach 1.50 × 10^−3^ S cm^−1^ with t_Li+_ exceeding 0.80 at room temperature, demonstrating that pore charge topology translates into high selectivity, and mesoscale void topology is preserved.

A more advanced topographical concept is gating in channels, achieved by developing multicationic molecular chains within a parent COF. COF channels are allyl‐functionalized in a gate‐controlled system to incorporate polymerized ionic‐liquid‐derived chains that extend to cover the pore walls, creating a percolating cationic network. As shown in Figure 7, these multi‐cationic chains serve as cation‐permeable gates, permitting the flow of Li^+^ but restricting anionic mobility by strong Coulombic interactions [25]. At a microscopic scale, the coordination metrics of Li–N (∼1.8 Å) and Li–O (∼1.9 Å) distances reflect the reshaping of the local binding environment within the restricted, charged pore space, linking the gate's topology to coordination‐driven transport.

**FIGURE 7:**
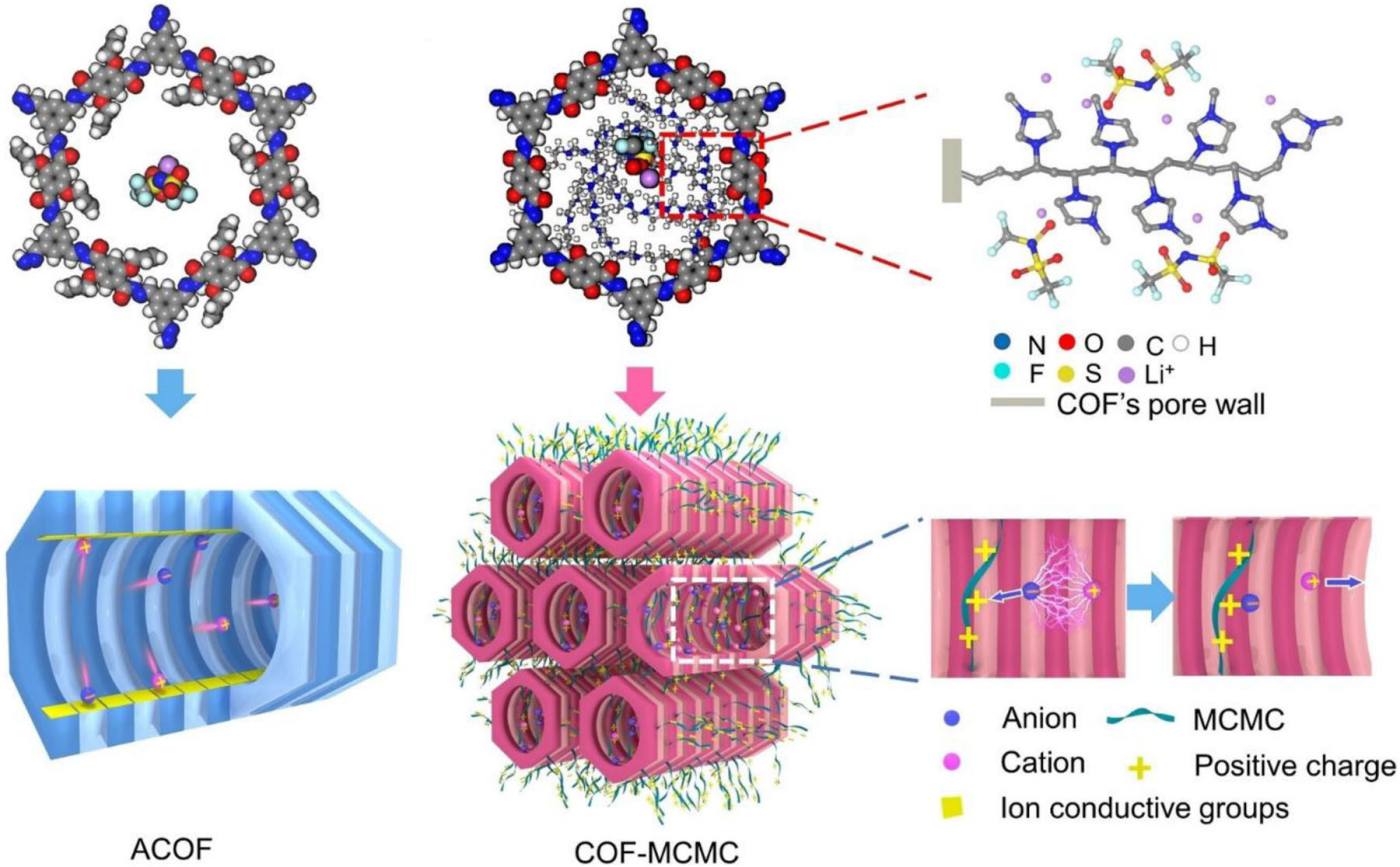
Concept of the COF‐MCMC design, in which multi‐cationic molecular chains act as Li^+^‐selective gates within COF channels, enabling gate‐controlled superionic conduction. Reproduced with permission [25].

Zwitterionic channel chemistry may be considered a step between anion‐ and cation‐fixed topologies. The local ion pairs formed by internal zwitterions in Zwitt‐COF can be used to dissociate salts and regulate Li^+^ solvation, while the pore structure maintains long‐range order. The topological benefit is that charge compensation is intrinsic to the structure, thereby reducing the importance of mobile counter‐ions that would otherwise cause polarization.

#### 4.1.6 3D COF Topologies: Interpenetration, Oriented Channels, and Robustness Against Misorientation

Although 2D COFs are primarily used in the electrolyte literature, 3D COFs offer certain topological advantages for practical batteries. To begin with, 3D connectivity eliminates the need to rely on a flawless texture: although crystallites may be randomly oriented, percolating void networks can be accessible in many directions. Second, 3D frameworks may be stiffer and more mechanically resistant to interlayer sliding, thereby maintaining channel continuity under pressure and cycling‐induced stress. Nevertheless, even random loading of 3D COF powders exhibits grain‐boundary constrictions and dead‐end pores; thus, achieving high σ_eff_ is usually still advantageous over densification or polymer/plastic‐crystal infiltration that fills interparticle joints.

Interpenetrated dia nets emphasize both opportunity and trade‐off. Lai and co‐workers formulated high‐degree‐of‐conjugation 3D COFs (3D‐SpCOF and 3D‐SpCOF‐OH) with I41/AMD symmetry, consisting of a sevenfold‐interpenetrated diamond topology (dia‐c7). The structures exhibit large unit cells (a = b = 35.554 Å and c = 11.351 Å), are permanently porous, and have a high surface area (≈1016 m^2^ g^−1^) (3D‐SpCOF). As illustrated in Figure 8, this synthetic design generates a 3D framework that can host a plastic‐crystal electrolyte within its continuous pore network. Upon incorporation of succinonitrile (SN) with LiTFSI into the interconnected 3D pores, ambient temperature ionic conductivities of 0.64 mS cm^−1^ (3D‐SpCOF/SN/LiTFSI) and 1.21 mS cm^−1^ (3D‐SpCOF‐OH/SN/LiTFSI) were obtained, with relatively high Li^+^ transference numbers (t_Li+_ = 0.70 and 0.64, respectively).

**FIGURE 8:**
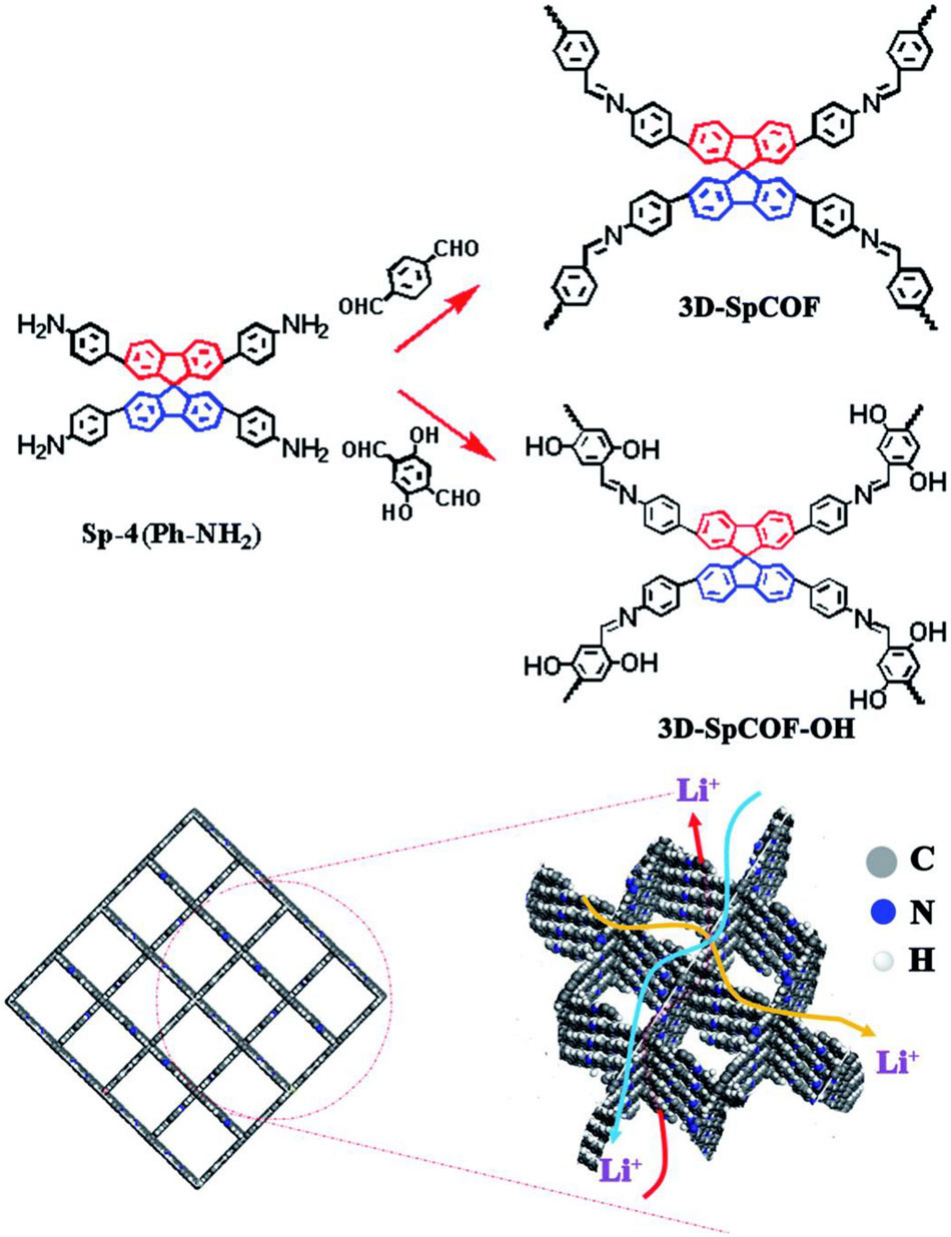
Synthetic design and 3D interpenetrated COF architecture (3D‐SpCOF series) for hosting a plastic‐crystal electrolyte, illustrating a 3D ion‐conduction scaffold. Reproduced with permission [61].

Topologically, 3D channel connectivity eliminates reliance on a perfect interlayer registry, which frequently limits 2D COFs, and offers alternative percolation pathways in the presence of grain boundaries or partial pore blockage. Simultaneously, interpenetration increases the lattice—desirable for mechanical integrity and macroscopic void‐defect prevention—but inexorably decreases the free volume of the lattice and can close apertures. Therefore, to achieve a high‐performance 3D COF electrolyte, aperture size and shape, the internal polarity/softness of salt dissociation, and the physical state of the guest phase must all be addressed together, as the 3D network needs to be a continuous, low‐tortuosity Li^+^ highway. Hence, there is a higher‐order interpenetration: moderate interpenetration can stabilize the scaffold and entrap anions, whereas overpenetration can shrink apertures, reduce available free volume, and create steric bottlenecks, thereby slowing Li^+^ diffusion.

Another strategy is diamond‐topology 3D COFs containing channel arrays of oriented rectangular shapes: the channels are oriented by embedding them within a 3D framework. TP‐COF/TB‐COF systems (diamond topology) are 1D channels that can be extended along the *c*‐axis, and the pore size can be tuned (e.g., ∼8.57 Å vs. ∼11.79 Å with structural modeling), and are also associated with distinct porosity (e.g., ∼62% vs. ∼77.2%). These findings support the idea that, in 3D COFs, channel engineering can still be achieved in a mechanically continuous topology, with the added advantage of reduced dependence on near‐perfect interlayer registry.

A systematic comparison of two‐dimensional (2D) and three‐dimensional (3D) covalent organic framework electrolytes across four design stages clarifies the conditions under which each dimensionality is advantageous. Regarding connectivity, layered 2D COFs provide wide (typically 1–5 nm), low‐tortuosity channels, but these channels are oriented only along the stacking direction. Consequently, device‐scale transport in 2D COFs is highly dependent on registry, texture, and alignment. In contrast, 3D networks enable multidirectional percolation that is inherently tolerant of crystallite misorientation, although this comes at the expense of aperture narrowing due to interpenetration and reduced free volume. In terms of coordination, the larger accessible pore volume of 2D COFs facilitates dense donor decoration and guest phase incorporation, whereas similar functionalization in interpenetrated 3D networks can introduce steric bottlenecks. With respect to coherence, 2Dframeworks are susceptible to stacking slip and turbostratic disorder, which can disrupt axial channels. Conversely, 3D covalent connectivity secures the pathway topology within the bond network and imparts greater mechanical robustness against interlayer sliding under stack pressure and cycling‐induced stress. Finally, in the context of hybridization, both dimensionalities benefit from mobile guest phases. However, available quantitative case studies remain asymmetric: the highest single‐component conductivities and transference numbers have been reported for 2D systems (e.g., 8.81 × 10^−3^ S cm^−1^ with t_Li_^+^ = 0.97 for the imidazolate Li^+^@PI‐TMEFB‐COF), Among the representative systems identified in the present literature survey involve hybrid systems (1.21 × 10^−3^ S cm^−1^ with t_Li_^+^ = 0.64 for 3D‐SpCOF‐OH/SN/LiTFSI). Notably, 2D/3D pairs that would isolate the effect of dimensionality alone have not yet been reported (Table 3).

**TABLE 3:** Quantitative comparison of representative COF‐based solid‐state electrolytes, linking structural descriptors to reported transport metrics.

Functionality (Descriptor)	Representative COF	Electrolyte class	Pore size(nm)	Staking	Crystallinity	RT ionic conductivity, σ (S cm^−1^)	E_a_ (eV)	Li^+^ transference number (t_Li+_)	Membrane form and Thinness	Key transport effect	Ref.
Multi‐cationic gate	COF‐MCMC	2D multi‐cationic gate	—	Eclipsed	Highly crystalline	4.9 × 10^−4^	0.31	0.71	Membrane (∼42 µm)	Li^+^ ‐selective gate‐controlled transport	[25]
Sulfonimide	Sulfonimide COF	2D anionic, single‐ion (quasi‐solid‐state)	∼0.27	Eclipsed, 2D	Highly crystalline	4.3 × 10^−4^	0.24	0.90	Pellet	Charge‐delocalized single‐ion transport	[44]
Neutral hybrid	TPBD/LiPF_6_/PEO	2D neutral, COF‐polymer hybrid	—	TPBD framework	Highly crystalline TPBD; reduced PEO crystallinity	5.43 × 10^−4^	0.37	—	Pellet (13 nm dia.)	Polymer‐assisted ion transport	[45]
Zwitterionic	PSZ‐COF	2D zwitterionic, eutectic IL‐phase hybrid	∼1.3–1.4	AA/AB1 (comparable)	Highly crystalline	1.27 × 10^−4^	0.15	0.62	Pellet	Salt dissociation with reduced anion mobility	[47]
Neutral hybrid	DMA@LiTFSI^−^mediated DLC	2D neutral, in situ polymerized hybrid	∼1.8	2D COF (eclipsed AA)	Highly crystalline	1.65 × 10^−4^	—	0.85	Membrane(∼32 µm)	In situ polymerized ion‐conduction network	[53]
3D interconnected plastic‐crystal hybrid	3D‐SpCOF‐OH/SN/LiTFSI	3D neutral, plastic‐crystal hybrid	—	3D interpenetrated framework	Highly crystalline	1.21 × 10^−3^	0.03	0.64	Pellet(∼130 µm)	3D ion‐conduction scaffold	[61]
Imidazolate	Li^+^PI^−^TMEFB‐COF	2D ionic, PC‐plasticized.	∼4.9	Eclipsed	Highly crystalline	8.8 × 10^−3^	0.41	0.97	Pellet	Charge‐delocalized single‐ion transport	[67]
Nitrile‐functionalized	CN‐iCOF	2D anionic (nitrile), solvent‐free	∼1.43/2.92	Eclipsed AA	Highly crystalline	1.33 × 10^−5^	0.20	0.93	Pellet	Enhanced Li^+^ coordination and salt dissociation	[71]
Phosphate transporter	TpMbh‐PO_3_Li_2_	phosphorylated COF	∼1	AB (staggered)	Highly crystalline	1.7 × 10^−3^	0.14	0.90	Membrane (40–50 µm)	Relay‐assisted Li^+^ hopping	[75]
Redox‐active (anthraquinone‐functionalized)	AQ‐Si‐COF	2D anionic, redox‐active	∼0.6/1.4	2D layered	Highly crystalline	9.8 × 10^−3^	0.09	0.92	Pellet	High single‐ion conduction	[76]

The lack of direct comparative data constitutes a significant research gap. Since existing 2D and 3D systems differ concurrently in linkage chemistry, pore size, and guest phases, current performance ranking cannot be conclusively attributed to dimensionality. Benchmarking 2D and 3D families constructed from identical monomer chemistries and characterized using the reporting toolkit described in Section 7.4 (including transport anisotropy and σ_eff_ at defined membrane thickness) is identified as a priority experiment. Such studies are essential for clarifying the trade‐off between the alignment sensitivity of 2D channels and the aperture and free‐volume limitations associated with 3Dinterpenetration.

#### 4.1.7 Hierarchical and Composite Topology: Percolation, Contact‐Region Pathways, and Continuous Ion Networks

COFs are seldom used in battery‐relevant applications as single crystals; they are employed as powders, pellets, thin films, or fillers in polymeric substrates. As a result, hierarchical topology, which is the connectivity of COF domains, polymer phases, and contact regions on the micrometer to millimeter scale, commonly prevails in device‐level performance despite unit‐cell topology being favorable [69].

One typical composite topology is the dispersed‐filler structure, in which COF particles are evenly dispersed within a polymer electrolyte. Even small COF loading (e.g., 3 wt%) in CPs‐BT/PEO composites is enough to form distributed porous domains to inhibit polymer crystallinity and aid fast‐ion conduction; the reported ionic conductivity is 1.5 × 10^−4^ S cm^−1^ at 40°C and indicates that even non‐percolating COF inclusions can enhance macroscopic transport by changing polymer topology and forming local shortcut pathways.

A similar structure is a dispersed‐filler topology that employs charged COFs to promote dissociation of the polymer–salt complex at the polymer–COF interface. In addition to the low‐additive EB‐COF‐modified PEO‐based electrolytes, low additive fractions raise ionic conductivity (the highest ionic conductivity is reported at 50°C, 0.19 ×10^−4^ S cm^−1^, in the case of an optimized loading), suggesting that the large internal surface area and porous topology of an additive generate a high‐volume contact‐region network of anions.

Vinylene‐bound COF additives also represent another example of the advantage of hierarchical topology. Introduction of VCOF‐1 (<4 wt% in PEO‐based solid polymer electrolytes) is claimed to enhance ionic conductivity and Li^+^ transference number by ∼25 and ∼46%, respectively, at 60°C, which is attributed to the scenario in which the cylindrical COF pores and polymer segments collectively form a cooperative pathway and minimize effective hopping distances in the composite. In this system, the connecting topology refers to the relationship between the polymer segments and the pores on the filler surface, rather than long‐range COF percolation.

The second type of composite topology is the membrane‐composite topology, in which COFs are embedded within continuous polymer membranes (e.g., via electrospinning) and subsequently densified. PEG‐COM membranes are an example of how such an architecture can couple unit‐cell channel topology with a continuous fiber network, thereby achieving ultrathin macroscopic dimensions (PEG2‐COM thickness ∼22 µm) and forming a low‐tortuosity composite path length along the separator direction [10].

The third composite topology is the in situ‐welded topology, which is formed by electrolyte‐mediated or in‐channel polymerization. Polymerization of the infiltrated electrolyte phase in electrolyte‐mediated LiCOF systems produces a mechanically engineered solid that eliminates grain‐boundary impedance while retaining pore‐directed ion transport, effectively transforming a granular topology into a welded percolation network. The same topological logic is employed in gate‐controlled COF systems, in which polymerized cationic chains are cultured in channels to form continuous internal conduction networks.

The fourth topology is the hollow or core–shell filler topology, in which internal empty spaces are used to rearrange polymer filling and ion distributions. Nitrogen sorption reveals a narrow pore size in CF_3_‐COF‐based hollow architectures (∼2.2 nm) with a BET surface area of ∼412.6 m^2^ g^−1^; the hysteresis behavior suggests that the shell pores are open to a hollow interior. When PVDF‐HFP is confined within and around these porous structures, it forms a confined interface that suppresses polymer crystallinity and limits the transport of large anions [69]. These architectures demonstrate that topology can be designed across multiple nested scales (from the pore to the shell to the hollow core) to enhance Li^+^ mobility and prevent anion migration.

#### 4.1.8 Computational Descriptors Linking Topology To Transport: Anisotropy, Tortuosity, and Energy Landscapes

Computational descriptors are becoming increasingly important, as quantitative correlations between topology and transport increasingly depend on descriptors that link channel geometry to activation barriers and ion distributions. Anisotropy: In layered COFs, one of the central quantities is anisotropy: the difference between migration barriers in the direction perpendicular to the channels and the basal plane. DFT calculations for PEG‐functionalized COFs indicate that the axial migration barriers (i.e., ∼0.56 and 0.43 eV) are significantly lower than the planar migration barriers (i.e., ∼1.02 and 1.31 eV), providing a mechanistic explanation for why an oriented channel topology is essential for high conductivity in membranes. These calculated barriers are supposed to be intrinsic tendencies in idealized channels; an apparent experimental E_a_ obtained in σ(T) is an effective quantity, which also represents salt dissociation, pore occupancy, and microstructural resistances; hence, qualitative agreement (trend/ranking) is likely to be even more common than quantitative matching.

An analogous perspective is offered by molecular dynamics, which tracks probability density and deduces free‐energy landscapes inside pores. Li^+^ probability density is found concentrated in the middle of the tunnel, not in proximity to the framework, in COF‐5 tunnels filled with Li salt and solvent, and Li^+^ diffusion events occur by concerted cleavage/formation of coordination bonds and anisotropic rotational dynamics of anions and solvent molecules, suggesting a 1D diffusion picture of a liquid. These simulations explain why a topology that allows continuous, low‐barrier tunnels is required but not sufficient: the internal coordination environment must also permit rapid rearrangement.

Topology descriptors can also be useful with 3D structures. Computed pore size and porosity (e.g., of diamond‐topology 3D COFs with rectangular channels: ∼8.57 A and ∼62% vs. ∼11.79 Å and ∼77.2%) in diamond‐topology 3D COFs have a structural basis to interpret variations in mean square displacement and activation energy obtained in molecular dynamics, attributing channel geometry to effective diffusion in polymer‐infiltrated composites. The preferred migration pathways of Li^+^ in confined electrolyte phases are determined by comparing energy barriers along crystallographic axes in interpenetrated dia‐c7 nets, revealing that 3D connectivity and interpenetration are the favored paths for Li^+^ migration through electrolyte confinement.

At the mesoscale, the tortuosity links the unit‐cell topology with macroscopic transport. In ideally oriented eclipsed channels (e.g., textured Q‐COF films), tortuosity is close to unity and is uniform along the film thickness. In contrast, in randomly oriented pellets, tortuosity increases due to grain boundaries and misaligned channels, thereby increasing the effective diffusion length and the contributions of contact impedance. This concept provides a rationalization for why orientation and densification strategies can yield outsized benefits, even when intrinsic channel conductivities are similar.

At the mesoscale, tortuosity can be a major source of σ_eff_ loss in pelletized or composite COF electrolytes due to misaligned channels and poorly fused grain boundaries, which increase the effective diffusion length and add contact‐region resistance. However, tortuosity is not the only limitation: incomplete salt dissociation, ion pairing in overly large pores, and channel occlusion by guests/polymers will also limit conductivity. Separating these contributions requires reporting both intrinsic descriptors (pore size, donor density, computed barriers) and microstructural descriptors (porosity/density, texture, and thickness‐scaled impedance) to distinguish intrinsic channel transport from processing‐limited σ_eff_.

#### 4.1.9 Topological Metrology and Reporting Considerations for COF Electrolytes

Given the importance of topology for transport, rigorous characterization and reporting of topological features are essential to developing predictive design rules. At the level of the unit cell and the stacking, powder x‐ray diffraction coupled to Pawley/Rietveld refinement is generally employed to limit the values of the lattice parameters, the net symmetry, as well as the stacking registry, as in the case of bnn‐topology COFs (P3 space group) or anion‐immobilized keto‐enamine COFs (pore size ∼1.63 nm, interlayer distance ∼0.34 nm).

However, diffraction alone may not fully resolve the functional pore topology, as different stacking registries and defect distributions can yield similar diffraction signatures, particularly in partly crystalline membranes. Complementary imaging (SEM/TEM) is therefore needed to assess mesoscale topography, including densification, grain‐boundary voids, and film thickness and continuity. For example, cross‐sectional imaging of ultrathin Q‐COF films (∼50 µm) and their densely stacked microstructure is critical to link observed conductivity and cycling stability to the intended oriented‐channel topology. In comparison, thickness control in electrospun PEG‐COMs (∼22 µm) is a necessary descriptor for comparing areal resistance across studies.

Finally, topology reporting in composites should include not only intrinsic COF pore metrics (surface area, pore size distribution, stacking order) but also hierarchical descriptors such as filler dispersion, percolation threshold (if present), degree of polymer infiltration, and contact‐region continuity. Hollow/core–shell systems (e.g., CF_3_‐COF) require an explicit description of shell porosity and the accessibility of the hollow interiors, since these nested topological features play a key role in hindering the transport of polymer building blocks and anions.

#### 4.1.10 Geometry: Topology‐Guided Design Principles and Outlook

Across the broad spectrum of COF electrolyte families, several principles emerge from topological analysis. First, transport can be achieved when a continuous pathway through the unit cell along the membrane thickness is maintained; this requires control of the stacking registry in 2D COFs and/or the use of mechanically continuous 3D nets that minimize the misorientation penalty. Second, pore size has to be co‐optimized with coordination chemistry: smaller than 2 nm pores (e.g., ∼1.50 nm VCOF‐1, ∼1.63 nm anion‐immobilized COFs) may favour selectivity and carrier density but require fast rearrangement of the coordination; larger pores (e.g., ∼6.85 nm CPs‐BT) will indeed be better suited as main polymer‐electrolyte modifiers than filtering single ions.

Third, the role of stacking and mesoscale orientation often becomes the crucial step to overcome and translate from “good” intrinsic channel topology to low‐resistance electrolytes: the textured thin films (Q‐COF) and ultrathin electrospun membranes (PEG‐COM) illustrate how reduction of tortuosity and thickness can have a huge impact on the practicality of the results due to enhanced effects of axial anisotropy. Fourth, charge topology provides excellent levers for selectivity by combining charge and pore topology: iCOF/PIL composites, anion‐immobilized COFs, the use of COFs with a charge twist (e.g., with a charge dipole), or COFs with a gate (COF or anion‐gated COF) all show that fixed charge or quasi‐fixed charge distributions may improve the Li^+^ transference and suppress the anion polarisation as long as pore accessibility is preserved.

Fifth, composite topology at contact boundaries may be as important as net topology anyway. Dispersed‐filler additives (CPs‐BT, EB‐COF, VCOF‐1) reconfigure polymer crystallinity and form contact‐region networks that modify ion distributions; electrolyte mediation and a hollow/core–shell architecture minimize grain‐boundary resistance and limit anion migration through controlled confinement. Overall, further development of COF electrolytes will require net design, stacking control, and mesoscale architecture to be integrated into a unified structure–topology–property correlation, with reported values of both intrinsic pore‐size measurements and hierarchical percolation indices, to achieve cross‐study comparability.

### 4.2 Chemical Functionality of Pore Walls

While framework topology determines the dimensionality and continuity of transport channels, the chemical functionality exposed on the pore walls in COFs governs the local ion–host interactions that ultimately determine the lithium‐salt dissociation equilibrium, the energetic landscape for Li^+^ hopping, and the extent of anion immobilization. Because COFs reveal periodically ordered functional groups with angstrom‐level precision, engineering the pore walls provides a direct path to converting geometrically defined nanochannels into ion‐selective, high‐transference‐number “ion highways” without segmental motion of the framework. Recent COF electrolytes thus put an ever‐growing focus on the modification of pore‐wall polarity and Lewis basicity/acidity through (i) neutral lithiophilic donor sites to emulate solvation, (ii) covalently tethered anions for single‐ion transport, and (iii) cationic or electrostatic sequestration of mobile anions and reconstruction of the ionic microenvironment [44]. Across the reported electrolyte compositions, the motivation for incorporating COF hierarchical structures was to increase Li^+^ carrier density and/or t_Li+_ by promoting greater salt dissociation and reducing anion mobility. Across the reported COF electrolytes, ether/oligoether motifs (often with confinement), carbonyl‐rich motifs, and fixed‐charge motifs (e.g., sulfonates/sulfonimides (anionic) or imidazolium/guanidinium (cationic)) have been most consistently shown to improve Li^+^ carriers’ density and/or t_Li+_ due to their ability to favour salt dissociation [55, 58, 67, 70, 71, 72, 73, 74, 75].

#### 4.2.1 Neutral Lithiophilic Donors and Solvation‐Mimetic Pore Surfaces

Neutral polar donors embedded in the pore walls (with ether oxygen atoms being the most prominent, along with N‐containing heterocycles) could thus mimic the coordination environment of liquid solvents/PEO while retaining the mechanical and structural benefits of a crystalline host. In this design, the transport of Li^+^ is facilitated by a series of reversible coordination–decoordination (“hopping”) reactions between neighboring donor sites along the channel wall, in which the coordination strength is high enough to dissociate the lithium salt but not so high as to trap Li^+^ and raise activation barriers.

A striking example of a donor‐site synergy is given by imidazolate COFs with methoxyethoxy side chains. Yuan et al. prepared PI‐TMEFB‐COFs using the Debus‐Radziszewski multicomponent process. They capped the framework with an imidazolate (iCOF) skeleton by ionizing it, then adding LiClO_4_ to yield Li^+^@PI‐TMEFB‐COFs. Control experiments revealed that removing the ether chains (Li^+^@PI‐TFB‐COFs) significantly restricted LiClO_4_ dissociation, while removing the ionic moieties (PI‐TMEFB‐COFs‐LiClO_4_) restricted ionic mobility. The coexistence of methoxyethoxy donors (salt‐dissociation function) and imidazolate sites (Li^+^ coordination/hopping sites) led to an optimized room‐temperature Li^+^ conductivity of 8.81 mS cm^−1^ and an extraordinarily high t_Li+_ of 0.974, consistent with near single‐ion transport behavior.

Beyond the use of side‐chain donors, electron‐rich heteroatom linkages in the COF backbone can act as “built‐in” lithiophilic sites. In PEO‐based composites with pyrazine‐linked (P‐COF) and imine‐linked (I‐COF) frameworks, both the polar pyrazine/imine functionalities were reported to interact strongly with Li^+^ to enhance salt dissociation and increase the room‐temperature ionic conductivity to 1.86 × 10^−3^ Scm^−1^. Although such composites have more than one ion‐transport contribution, these results support a more general principle governing ion transport in the pore walls: dense, spatially distributed Lewis‐basic sites can reduce the entropic/enthalpic penalties associated with Li^+^ migration by offering multiple coordination junctures along the ion‐transport direction (Table 3).

Carbonyl‐rich frameworks offer an alternative donor landscape, in which closely packed C═O groups in the pore walls serve as frequent coordination sites for Li^+^. In polyimide‐based COFs, imide carbonyls have been used to introduce strong dipoles and a high local density of hard oxygen donors, thereby promoting a strong dielectric response in confined channels and facilitating the dissociation of lithium salts (without relying on soft, mobile side chains). In addition, Liu et al. reported the polyimide COF (TAPA‐PDI‐COF) and fabricated solvent‐free solid‐state electrolytes by inserting a plastic‐crystalline succinonitrile (SN) phase containing LiTFSI into aligned channels (LiTFSI@SN@COF). As shown in Figure 9, the TAPA‐PDI‐COF framework and the confined LiTFSI/SN generate cooperative C═O (COF) and C≡N (SN) coordination, which is associated with anion motion restriction but with rapid Li^+^ ion exchange between adjacent donors.

**FIGURE 9:**
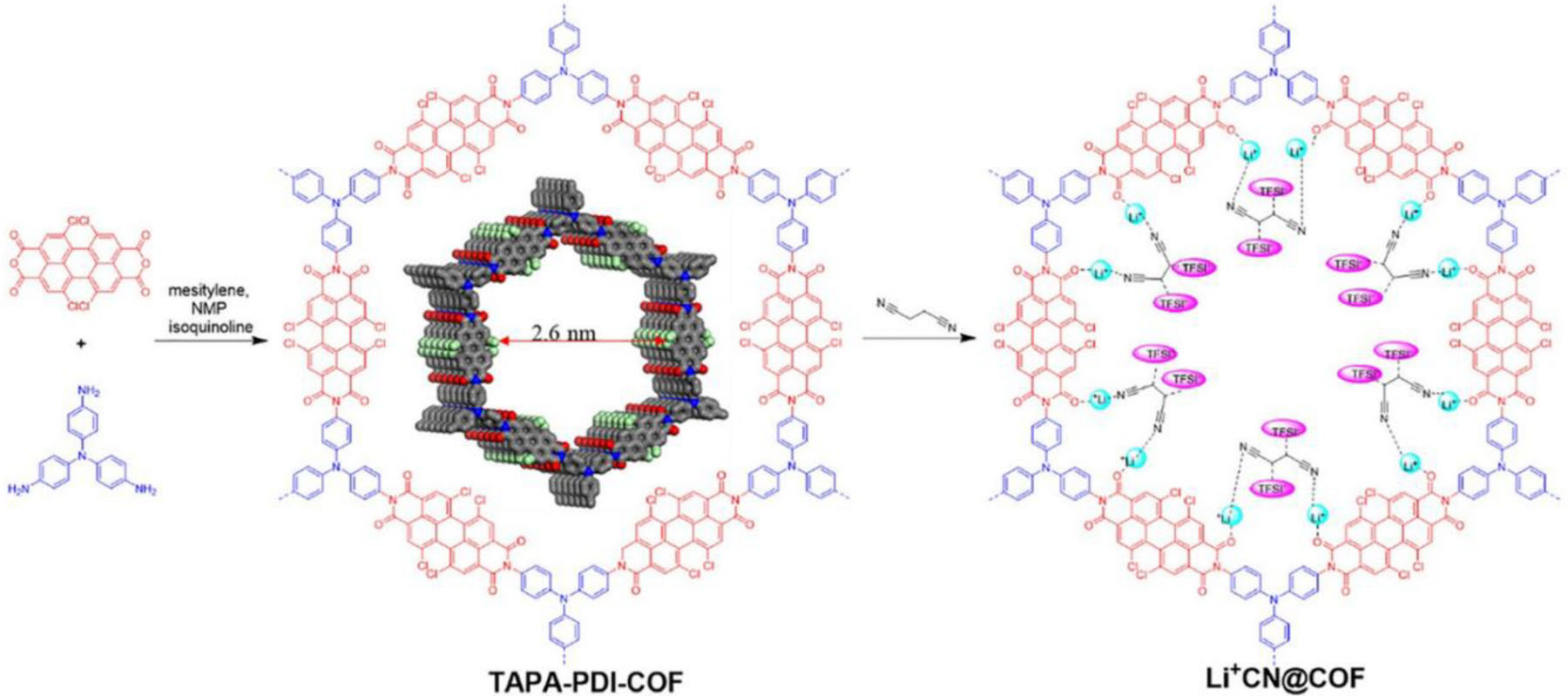
Polyimide COF (TAPA‐PDI‐COF) with carbonyl‐rich pore walls and the incorporation of LiTFSI and succinonitrile within aligned channels, generating cooperative C═O/C≡N coordination environments for Li^+^ transport. Reproduced with permission [74].

Electrochemically, LiTFSI@SN@COF exhibited an ionic conductivity of 1.02 × 10^−3^ S cm^−1^ at 80°C, a Li^+^ transference number of 0.855 at room temperature, and a low activation energy of 0.18 eV. Density functional theory analysis also suggested that Li^+^ migration was favored along in‐plane transport paths with short hopping distances compared with the axial direction, indicating that the spatial distribution of pore‐wall donors in layered COFs could be used to guide the preferred migration path by periodically distributing Li^+^ sites in the framework plane. An important caveat is that very strong Lewis basic sites may form deep binding wells, leading to an increased E_a_ due to trapping of Li^+^; hence, optimal pore wall donors are a compromise between high dissociation (carrier density) and sufficiently labile coordination to achieve rapid site‐to‐site exchange.

#### 4.2.2 Anionic Pore‐Wall Functionalities for Single‐Ion Conduction

Covalently anchoring anions onto pore walls is a known method for enhancing Li^+^ transport and alleviating concentration polarisation in solid‐state electrolytes. In such single‐ion COFs, the pore‐wall anion is responsible for the Li^+^ carrier density and for modulating Li^+^ mobility via its Lewis basic character (“hardness/softness”) and charge delocalization. As discussed by Choi et al., Li^+^, being a hard Lewis acid, displays strong binding with hard bases such as carboxylate (─CO^2−^), which leads to slow transfer of Li^+,^ whereas softer sulfonate (─SO^3−^) and more delocalized fluorinated sulfonimide motifs weaken binding with Li^+^ and give way to faster Li^+^ hopping at room temperature.

This reasoning about hardness–softness is evident in the early designs of solution‐processable COF electrolytes based on a hydrazone framework (Li et al.). The rigidity of the COF is attributed to ordered one‐dimensional channels. At the same time, the pore chemistry is responsible for low‐temperature conduction: the hydrazone COF electrolyte exhibited a conductivity of 10^−5^ S cm^−1^ at −40°C, with a [0.92] Li^+^ transference number. The combination of crystallinity and chemically addressable pore walls also makes hydrazone COFs suitable for post‐synthetic modifications, which may provide a practical route toward systematic tuning of the anionic microenvironment within the channels.

More recently, sulfonimide‐functionalized COFs have been proposed as a new generation of quasi‐solid single‐ion electrolytes. Wu et al. prepared crystalline COFs that contain sulfonimide functional groups, in which a combination of the delocalized anionic charge and a preferred transport pathway yields a room‐temperature lithium‐ion conductivity of 4.3 × 10^−4^ S cm^−1^ at 298 K, with a low activation energy of 0.24 eV and a high lithium transference number of ≈0.90. Importantly, the authors noted that coordination of strong electron‐withdrawing and charge‐delocalizing substituents promoted Li^+^ transport, highlighting the interplay between local electrostatics and anion softness in controlling Li^+^ dissociation and hopping dynamics in confined COF channels.

An instructive extension of anion design is the development of electronically engineered single‐ion COFs that couple anionic charge to a coordination landscape featuring nitrile‐rich features. An azanide‐based single‐ion COF (CN‐iCOF) was achieved by cyanations via a Strecker reaction, converting a reversible imine linkage into a more stable sp^3^‐carbon‐containing azanide anion. DFT and molecular dynamics studies found that the electron‐withdrawing nitrile groups decrease the HOMO energy and increase Li^+^ dissociation due to charge delocalization to allow high t_Li+_ (0.93) and high oxidative stability up to 5.6 V (vs. Li^+^/ Li) to be achieved simultaneously [17]. Moreover, the motifs of the aligned nitrile form contorted “migration ladders” down the length of one‐dimensional anionic channels reducing the calculated Li^+^ migration barrier (e.g., from 8.99 to 4.38 kcal mol^−1^) leading to ambient conductivity of 1.33×10^−5^ S cm^−1^ with no liquid/plasticizer additives; as shown in Figure 10a, nitrile‐rich pore walls and the anionic framework establish a directional Li^+^ transport that promotes rapid migration and improve high‐voltage performance.

**FIGURE 10:**
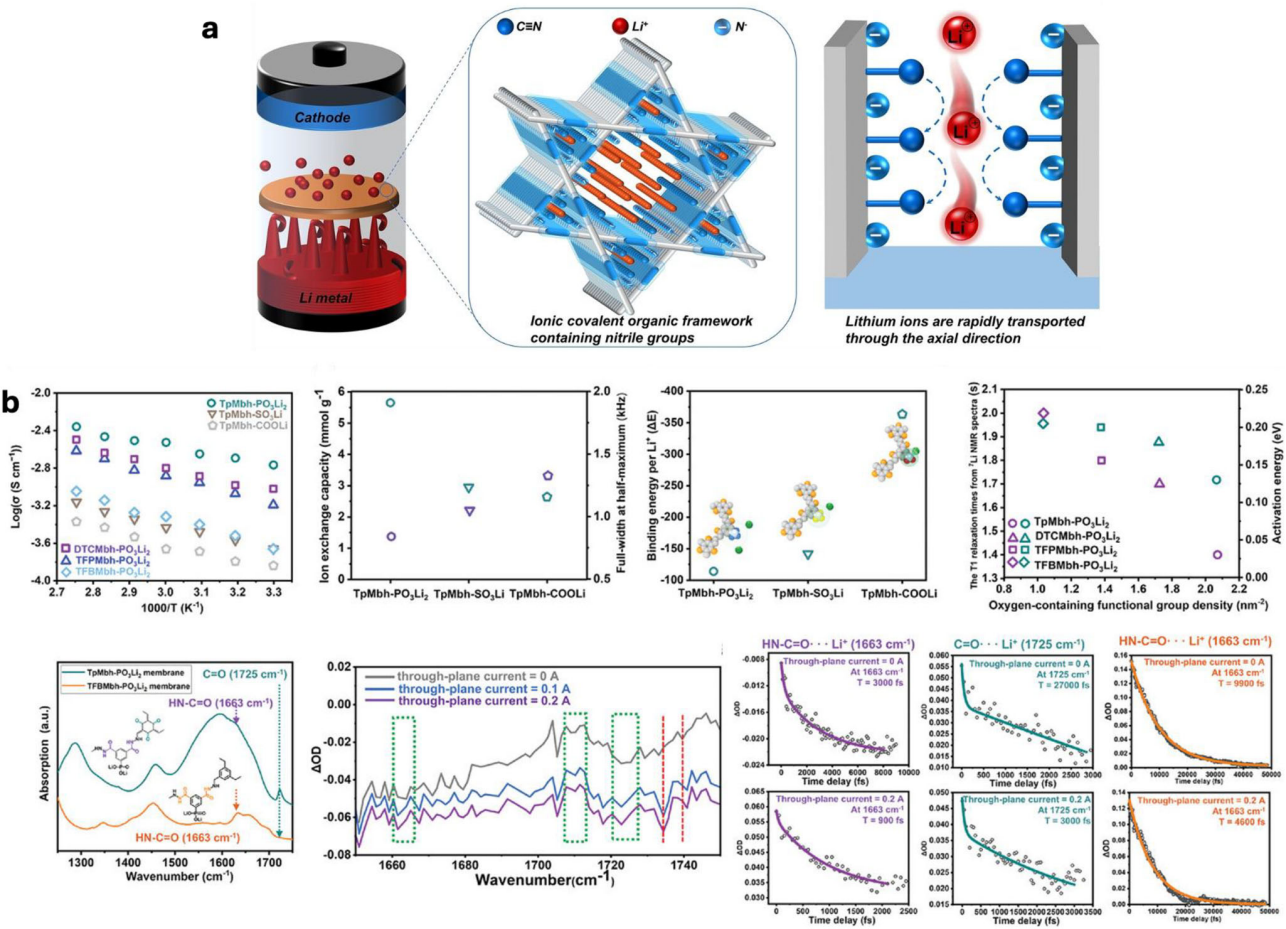
(a) Conceptual representation of nitrile migration “ladders” in azanide‐based single‐ion COF (CN‐iCOF), where nitrile‐rich pore‐wall coordination sites and anionic backbones synergistically facilitate axial Li^+^ transport and high‐voltage tolerance. Reproduced with permission [71] (b) Synergistic transporter/co‐transporter effect in phosphorylated ionic COF membranes; comparison of phosphate‐, sulfonate‐, and carboxylate‐based transporters; and illustration of the influence of co‐transporter oxygen density on Li^+^ conductivity and activation barriers. Reproduced with permission [75].

A different way of thinking about the design of anionic pore walls is suggested by membranes based on ionic COFs, in which the immobilized anion is explicitly considered an agent for transporting Li^+^ rather than a counterion. Pang et al. used crystalline ionic COF nanosheets with three types of anionic transporters (phosphate, sulfonate, and carboxylate; ─PO_3_^2−^, ─SO^3−^, and ─COO^−^) and assembled them into dense membranes with AB‐stacked lamellae. In this architecture, the binding capacity and release kinetics of Li^+^ are controlled by the transporter's anions. In contrast, neighboring oxygen‐containing groups within the framework are Li^+^ co‐transporters, providing relay sites between the neighboring transporters so that they can quickly hop [80].

By varying the identity of the transporter and the density of co‐transporter oxygen sites, the authors formulated a binding‐releasing‐hopping mechanism that couples local electrostatics to macroscopic conductivity. As shown in Figure 10b, phosphate‐based membranes (TpMbh‐PO_3_Li_2_) achieved a room‐temperature Li^+^ conductivity of 1.7 mS cm^−1^ with a Li^+^ transference number of 0.90; with increasing clotting co‐transporter density, the Li^+^ transport activation energy (down to ∼0.14 eV) decreased, consistent with an energy landscape in which the proximity of relay donors flattens. These results indicate that the optimal single‐ion pore‐wall chemistry is not determined by anion softness alone; rather, fast transport can be realized by combining a fixed‐charge site with high carrier density and kinetically labile coordination in a channel environment enriched with neutral relay donors.

#### 4.2.3 Cationic Pore Walls and Electrostatic Anion Sequestration

Instead of covalently tethering anions to fixed positions, another approach to immobilizing anions in pores is to incorporate positively charged sites into the framework of the separatory membrane, which electrostatically trap counter‐anions within the pores. Such cationic COFs can produce highly polar nano‐environments that favor the dissociation of lithium salts and, in principle, enable ion‐selective transport when anion mobility is suppressed by Coulombic binding and confinement. However, if anion sequestration is too strong (excessive charge density and/or pores that are too tight), anions can become statically pinned. At the same time, a strongly associated local salt phase forms, which is known to slow Li^+^ exchange, so the properties of cationic charge density and pore geometry should be balanced to immobilize anions without generating crowded, slow‐dynamics ion clusters.

Li et al. built an imidazolium‐based cationic COF (Im‐COF‐Br) and performed ion exchange to replace Br^−^ with bis(trifluoromethylsulfonyl)imide (TFSI^−^), yielding Im‐COF‐TFSI with a large observed pore size of 5.57 nm. A weakly coordinating, charge‐delocalized TFSI‐ counterion is favorable for lithium‐ion transport in charged frameworks, and Im‐COF‐TFSI‐based electrolytes achieved lithium‐ion conductivities of up to 4.64 × 10^−4^ and 4.04 × 10^−3^ S cm^−1^ at 353 and 423 K, respectively. These observations highlight that, even within the sphere of cationic channels, if a soft, delocalized counter‐anion can be selected, it is possible to minimize ion pairing while maintaining a high population of mobile carriers, namely Li^+^ ions.

Beyond a uniform electrical charge on the pore walls, “electrostatic gating” can be achieved by introducing cationic species as discrete binding sites periodically spaced along the channel. Gong et al. used multi‐cationic molecular chains (MCMC) within the pores of a COF to realize a cation‐selective gate that traps anions and facilitates Li^+^ transport through the channel without requiring segmental motion of the polymer. Such architectures highlight how continuous or discrete charge patterning of the pore walls can control the interplay among anion confinement, Li^+^ dissociation, and the percolation of transport pathways in COF‐based solid electrolytes.

#### 4.2.4 Zwitterionic Pore Walls and Internal Charge Separation

Zwitterionic motifs are a chemically integrated approach to simultaneously impart positive and negative charges to the pore surface, often producing an “ionic‐liquid‐like” polarity without requiring mobile counterions. In a Zwitterionic COF electrolyte (Zwitt‐COF), Kang et al. introduced 2‐(pyridinium‐1‐yl) acetate groups into the framework. These groups interact strongly with the Li salt, promoting its dissociation and reorganizing the ion‐transport channels, resulting in high ionic conductivity and a higher lithium transference number than in analogous non‐zwitterionic hosts. From the point of view of a pore wall, the presence of the so‐called Zwitterions can be regarded as the formation of built‐in dipoles/charge‐separated sites, which favor a high local dielectric response and allow multiple coordination motifs for Li^+^ while reducing the long‐range migration of anions. Because the overall electroneutrality of the frameworks (zwitterionic frameworks) may reduce the exchange of counterions and limit electrostatic swelling to uncharged vs. charged backbones, this is a great advantage. However, the long‐term stability under Li‐metal contact and cycling during charge/discharge remains a relatively unexplored research area, and systematic comparisons with analogous fully ionic COFs are required.

#### 4.2.5 Defect‐Enabled Post‐Functionalization and Cooperation Effects

In practice, to maximize the impact of pore‐wall chemistry, the density and chemical diversity of functional sites sometimes need to be increased without sacrificing crystallinity. Defect engineering provides a particularly useful handle by providing anchoring sites for post‐synthetic modification. Mi et al. synthesized a family of defective [3+3] COFs that contain active amine/aldehyde sites. Subsequently, they placed charged functional molecules into the pore walls via so‐called forward reactions (Schiff base reactions) and ion exchange. By the combination of well‐defined ion channels and an anionic sulfonate framework and electron‐withdrawing triazine units, the obtained COF‐ECJTU1‐60‐SO_3_Li‐based electrolyte exhibited a lithium‐ion conductivity of 1.30 × 10^−5^ S cm^−1^, a low activation energy of 0.22 eV, and a high t_Li+_ value of 0.87 at 303 K. Performance improvements were attributed in reported studies with a synergistic effect of ionic group and electron‐withdrawing units modulating both Li^+^ carrier density and dissociation/hopping energetics in the channels. A good rule of thumb is that too many post‐functionalizations or bulky moieties may disrupt stacking and reduce crystallinity/texture, which may offset the increased gain from added donor density (due to increased tortuosity and grain‐boundary resistance).

Cooperative electronic effects may also be exploited by Lewis‐acidic pore‐wall sites, which preferentially bind anions. In an in situ composite strategy of the combination of a B‐containing carbon–organic framework (COF) and a modified polymer electrolyte, Guan et al. attributed the enhanced dissociation of lithium salts to the synergy of the strong polar groups in the polymer chains and boron centers in the heptacyclic C–C framework of the COF, which enhanced the adsorption of the anions and increased the Li^+^ transference number from 0.28 to 0.51. While the mechanism of ion transport requires a detailed understanding of the composite microstructure, the present research strengthens a transferable pore‐wall design concept, i.e., the concurrent introduction of complementary Lewis acid/Lewis base sites allows the dissociation equilibrium to be shifted in favour of free Li^+^ by selective stabilization of the anions in the confined environment.

#### 4.2.6 Redox‐Active Pore Walls and Dynamic Li^+^ Exchange

Beyond static Lewis‐base coordination, redox‐active motifs incorporated into the pore walls of COFs can introduce electronically tunable Li^+^ binding/release pathways, thereby enhancing the effective carrier density and introducing new low‐barrier exchange sites. Li et al. reported an anthraquinone‐based CCOF (AQ‐Si‐COF) by introducing redox‐active anthraquinone units into an anionic silicate backbone. The material showed a room‐temperature conductivity of 9.8 mS cm^−1^ and a single‐ion transference number of 0.92, and the authors attributed this difference to multi‐Li^+^‐ (nine‐Li^+^) mediated charge/discharge processing coupled with Li^+^ complexation around the anthraquinone sites. Although redox‐assisted transport may obfuscate the distinction between an ion conductor and a charge‐storage host, the principle of design of the corresponding pore walls is instructive: pairing of both fixed‐charge sites and reversible redox centers may lead to a dynamic coordination landscape in which dissociation and hopping of Li^+^ are determined both by coordination chemistry and the redox state of the host framework [76]. Because such motifs are likely to undergo repeated redox fluctuations during battery operation, the reversibility of Li^+^ binding and the chemical robustness of the motif must be demonstrated over long‐term cycling to rule out irreversible structural changes, side reactions, or loss of crystallinity.

Collectively, these studies make pore‐wall chemistry a core design control for COF electrolytes. Neutral donor sites (ethers, imines, pyrazines, azoles) mainly control the solvation and hopping rate of Li^+^, anionic motifs (sulfonate/sulfonimide/azanide) control the single‐ion character and need to be optimized in terms of proper softness and charge delocalization, whereas cationic/zwitterionic motifs control the local confinement of anions and the local dielectric response. In the future, combining various functionalities in a spatial‐programming fashion, e.g., dissociation‐promoting donors and aligned hopping sites with electronically stabilized single‐ion backbones, seems especially promising for constructing room‐temperature, high‐voltage COF ion highways for next‐generation solid‐state lithium batteries.

### 4.3 Crystallinity

Crystallinity is the defining attribute of COF electrolytes, as it governs the coherence of the ion‐highway architecture presented in Sections 4.1and 4.2. While the geometric corridor and the local coordination landscape are defined by topology and pore‐wall chemistry, crystallinity plays a major role in determining whether these motifs repeat regularly and whether they are connected over macroscopic transport lengths. In effect, “crystallinity” in COF‐based solid‐state electrolytes is a multiscale quantity that spans unit‐cell order, stacking coherence in layered structures, crystallite‐size distributions, and the extent to which crystalline scaffold order is maintained upon salt loading, pore‐wall functionalization, or polymer infiltration. Recent views for ionic COF electrolytes have been focused on the notion that high crystallinity is a double‐edged characteristic: on the one hand, it forms a set of clearly defined ion highways, but on the other, highly ordered crystallites can be mechanically rigid, making it difficult to move from a crystalline powder to a dense, mechanically robust membrane and increasing the contact resistance as well if the microstructure is not intentionally designed [40]. However, far from being monotonic, the crystallinity‐transport relationship is nonlinear. While a higher level of order improves channel definition and minimizes static disorder, an overly rigid host–guest arrangement can also reduce local configurational freedom (primarily in polymer‐filled pores) and increase the residence time of Li^+^ ions; thus, there can be an optimal “coherence window” for a given occupancy state.

#### 4.3.1 Crystallinity as a Transport Design Variable

At the molecular level, crystalline order imposes constraints on the distribution of coordination sites, dielectric environments, and free volume that govern Li‐salt dissociation and Li^+^ migration. In the idealized form of a crystalline COF, ion hopping can be described as migration over a periodic potential, allowing structure–transport correlations to be elucidated and predictive modeling to be performed. However, real COF electrolytes are polycrystalline and are full of defects: stacking faults, grain‐boundary regions, and incompleteness of condensation can render channel continuity discontinuous, widen the distribution of site‐to‐site hopping distances, and cause transport bottlenecks that are not evident from unit‐cell models only [38]. Importantly, the directionality of ion transport in COFs implies that crystallinity is not only a binary (high vs. low) descriptor but also an anisotropic one, depending on registry and coherence along the channel axis.

#### 4.3.2 Metrology of Crystallinity in COF Electrolytes

Powder x‐ray diffraction (PXRD) remains the most commonly used method for measuring the crystallinity of the framework, stacking registry, and crystallite coherence. For 2D COFs, low‐angle reflections are reported to reveal in‐plane periodicity (e.g., (100)/(110)), whereas higher‐angle features can indicate interlayer stacking (e.g., (001)) and yield the interlayer spacing. Peak‐breadth and intensity changes can be particularly important in diagnosis: broadening generally implies diminished coherence lengths (e.g., crystallite size, defect concentration, or stacking disorder), while systematic shifts may indicate lattice expansion/contraction or ion uptake. For example, as shown in Figure 11, for cyano‐substituted COFs synthesized via a Knoevenagel reaction, the resulting PXRD pattern displays a diffraction peak at 2θ ≈ 3°, which is assigned to the (100) plane, and the Pawley refinement is compatible with an eclipsed AA stacking model [40]. Coupling PXRD with structural simulations (AA/AB stacking hypotheses) and Pawley/Rietveld refinement to local probes such as TEM/SAED provides a more rigorous picture of crystallinity, especially for ion‐conducting COFs, in which ionic functionalization and guest molecules can blur diffraction features [77].

**FIGURE 11:**
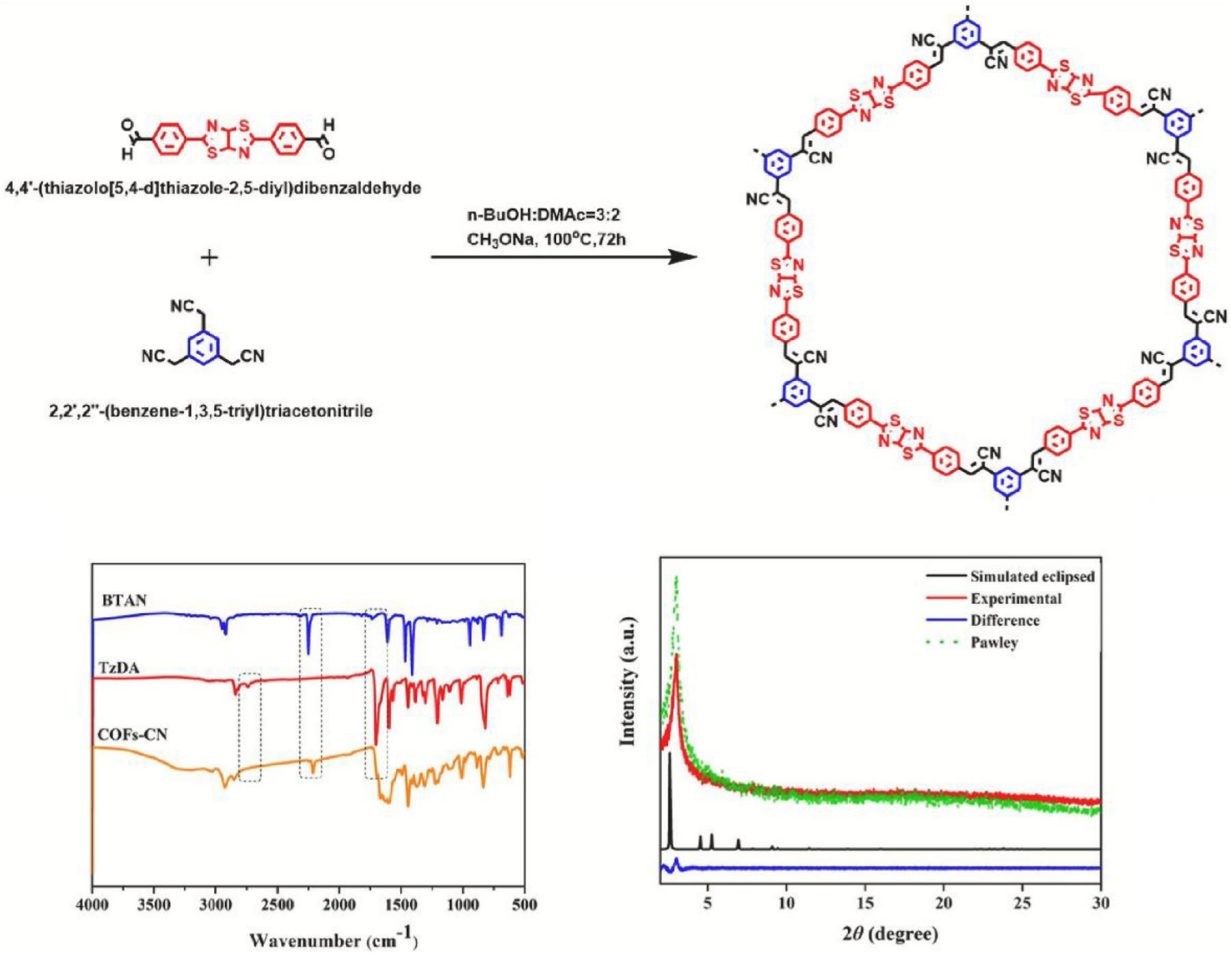
PXRD analysis, Pawley refinement, and simulated eclipsed AA‐stacking model used to evaluate crystallinity in a cyano‐substituted COF (COFs‑CN) processed into porous composite films. Reproduced with permission [40].

#### 4.3.3 Crystallinity Modulation in Ionic Functionalization and Post‐Synthetic Conversion

A recurring observation in ion‐conducting COFs is that incorporating ionic groups and flexible solvating side chains can partially reduce crystallinity, even when the periodic framework backbone is preserved. This effect is illustrated by the pyridinium COF electrolyte system reported by Lee and co‐workers. As shown in Figure 12a,b, the pristine pyridine‐rich COF (PCOF) exhibits a relatively compact particle morphology and clearly resolved lattice domains, indicating a well‐ordered layered structure. The corresponding PXRD pattern (Figure 12e) shows characteristic low‐angle reflections at 2θ = 3.10° and 7.35°, corresponding to the (100) and (200) planes, respectively [77]. A higher‐angle peak at 2θ = 23.1° was attributed to (001) stacking, yielding an interlayer spacing of 3.85 Å.

**FIGURE 12:**
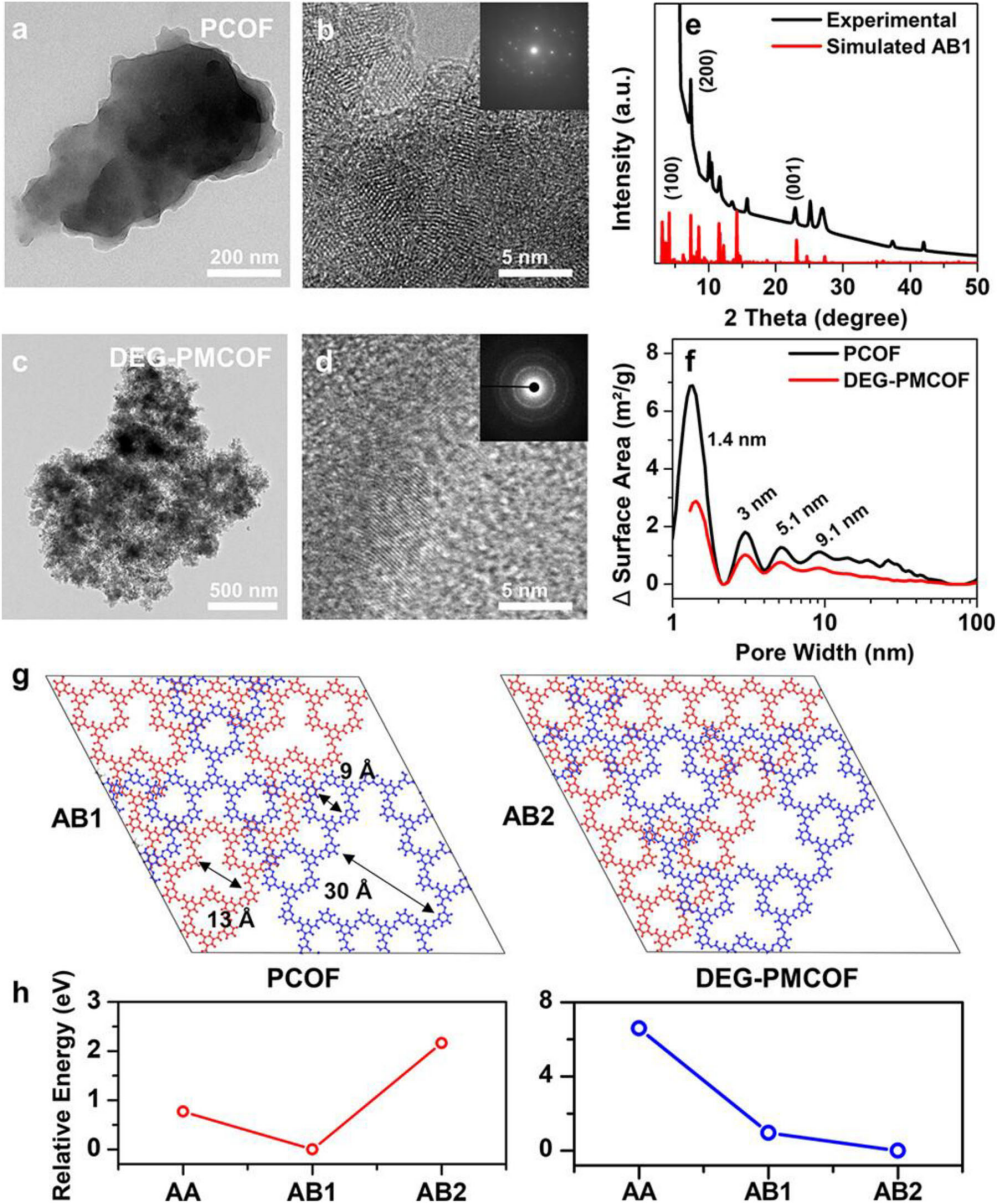
Post‐functionalization‐induced changes in crystallinity and stacking evolution in pyridinium COF (PCOF) and DEG‑PMCOF electrolytes, including experimental/simulated XRD patterns together with corresponding stacking models. Reproduced with permission [77].

Upon post‐functionalization to introduce diethylene glycol‐modified pyridinium groups (DEG‐PMCOF), the framework becomes more puckered and exhibits decreased crystallinity, consistent with the tendency of post‐synthetic conversion to broaden diffraction features, a widely observed phenomenon. In Figure 12c,d, the particles appear more compact than those in PCOF, while the HRTEM and SAED patterns indicate a modification of the lattice coherence. Crucially, BET analysis was used to prove that periodic pore distributions could be maintained despite the widening of diffraction peaks, which highlights the existence of a practical difference between (i) loss of long‐range coherence and (ii) breakdown of the intrinsic channel architecture (Figure 12f). According to the structural and relative energy profile analyses in Figure 12g,h, DEG functionalization shifts the most stable stacking mode from AB1 to AB2, highlighting its impact on interlayer organization and the pore environment.

From a design point of view, the crystallinity penalty due to functionalization may be avoided by choosing linkage chemistries and reaction conditions that maximize error correction, by limiting functionalization to spatially distributed defect sites, or by using stepwise protocols that do not alter the rigid backbone while appending mobile ion‐solvating segments. Mechanistically, moderate peak broadening need not correspond to poorer ion transport: partial disorder leads to enhanced local free volume and softening of the coordination environment, which may decrease the energetic cost of Li^+^ desolvation/re‐solvation as it moves from one binding site to another. The main danger is over‐amorphization, which can disconnect the channels and change the transport mechanism from a deterministic “channel” mechanism to a tortuous percolation path governed by boundary crossings.

#### 4.3.4 Balancing Framework Crystallinity With Polymer‐Phase Crystallinity in Composite Electrolytes

In many practical formats for solid‐state electrolytes, a crystalline COF scaffold is coupled with a polymeric ion conductor. In such systems, crystallinity would need to be optimized in both components. Whereas the COF requires sufficient order to maintain channel continuity, the polymer phase would often benefit from reduced crystallinity to enhance segmental mobility and increase the amorphous fraction, thereby improving Li^+^ transport. Shen and co‐workers demonstrated this duality by using a Lewis‐basic, nitrogen‐rich COF (N‐COF) as a functional scaffold for a flexible PEO‐based solid‐state electrolyte. The N‐COF itself exhibits a crystalline diffraction pattern with reflections attributed to several facets [e.g., (110), (101), (111), (202), (121), (220)], and the experimental XRD pattern is consistent with a proposed stacking model [77]. When used as a Li^+^‐loaded additive in PEO, the PEO‐Li^+^@N‐COF films exhibit broader, weaker PEO reflections at 2θ ≈ 19° and 23°, indicating low crystallinity in the PEO matrix. As shown in Figure 13, the COF framework largely retains its crystallinity. In contrast, disruption of polymer chain ordering by the porous scaffold increases the polymer's amorphous fraction, thereby accelerating Li^+^ motion within the polymer phase.

**FIGURE 13:**
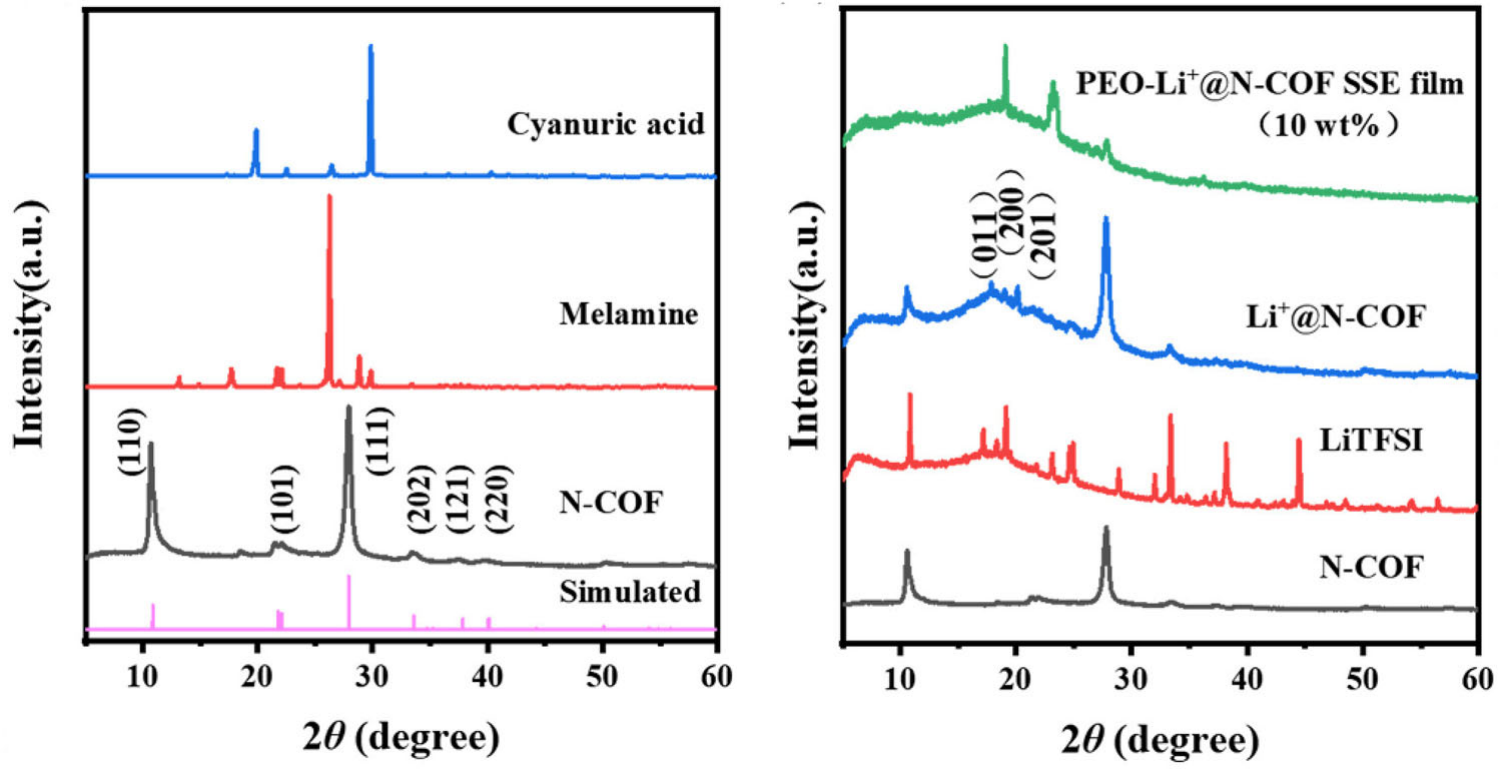
XRD patterns of N‐COF and Li+@N–COF/PEO composites, illustrating the retention of the crystalline framework and the suppression of polymer crystallinity in a flexible COF‐enabled solid‐state electrolyte. Reproduced with permission [78].

Phase‐inversion‐derived porous COF composite films give a complementary processing‐driven illustration. Wang and co‐workers reported that COFs‐CN are intrinsically crystalline and are obtained as an insoluble, infusible powder, thereby motivating the use of polymer‐matrix processing to produce scalable membranes. In their work, verification of crystallinity was based on PXRD and comparison with a simulated model of AA stacking; they also associated the crystalline stacking motif with channel dimensions (≈36 Å pore diameter and ≈4.0 Å interlayer distance) based on structural models. The broader implication for COF electrolytes is that crystallinity management is inseparable from processability: highly ordered COFs may require composite‐ or templated‐processing routes to transfer powder crystallinity to a continuous membrane without channel incoherence.

#### 4.3.5 Crystal Size, Grain Boundary Effects, and the Path Toward Channel‐Coherent Membranes

Beyond “how crystalline” a COF is, the distribution of crystal size and the density of grain boundaries can play a decisive role in the macroscopic conductivity. Small crystallites and powders that are not well fused can introduce numerous boundary crossings for migrating ions, thereby increasing tortuosity and contact resistance, even if intrinsic transport within channels is rapid. Strategies to enlarge crystallite size and approach single‐crystal‐like domains thus represent an underused avenue to enhance transport fidelity. Kim and co‐workers emphasized that the synthesis of large (≫µm‐scale) single‐crystalline 2D COFs is still challenging, but that seeded growth and the prearrangement of monomers using solvents can be employed to increase the crystal sizes and improve the crystallinity. They further pointed out that polymerization variables, such as monomer concentration, modulator loading, and temperature, can increase the size of the lateral crystals by as much as tens of times (reported to be ∼20 µm), and hence lead to smaller intraparticle disorder and reduced transport limitations from the remaining grain‐boundary area. In practice, the two techniques of improving stacking registry (intra‐crystal axial coherence) and increasing crystal size/densification (grain‐boundary density) are complementary because stacking order determines the intrinsic axial barrier within domains and crystal size and pellet microstructure determine the frequency/penalty of boundary crossings that control σ_eff_. The type of dominant lever will depend on whether σ is limited by intra‐domain hopping or resistance between particles.

Finally, it is worth emphasizing that “the polymer may cause lower crystallinity” in the electrolyte, not in the COF scaffold. In polymer‐dominant conductors, more than a dozen studies (including non‐COF composite polymer electrolytes) have revealed that hindering PEO crystallinity and favoring amorphous condensed chain packings can open numerous swift Li^+^ transport channels and augment room‐temperature conductivity [79]. Although the structural levers differ, the transference principle is identical: crystallinity must be designed to maintain continuous ion pathways while preserving the local dynamics necessary for rapid Li^+^ exchange between coordination sites.

#### 4.3.6 Integration and Reporting Recommendations

Looking into the future, crystallinity engineering should be considered alongside topology and pore‐wall chemistry, rather than regarded as an independent measure of materials. In the case of high‐performing COF electrolytes, crystallinity is better considered as essentially the structural bandwidth that influences how well a designed ion‐conduction motif (aligned channels, periodic hopping sites, anion immobilization patterns) is translated from a molecular blueprint into a membrane‐scale conductor. Reporting of best practice should therefore involve (i) PXRD peak assignments and refinement parameters (when possible), (ii) explicit stacking models and this evidence, (iii) crystallite size/orientation descriptors (especially of films), and (iv) polymer phase crystallinity parameters (DSC enthalpy, XRD peak breadth) in composite systems. This type of crystallinity metrology will be essential for establishing quantitative structure–activity relationships and enabling the rational design of COF ion highways for solid‐state lithium batteries.

## 5 Hybrid COFs

Hybridization has become a viable compromise approach for implementing crystalline COF “ion highways” as mechanically coherent, processable solid electrolytes that can be prepared as thin films, self‐standing membranes, or dense composite layers. In hybrid COF electrolytes, an ordered framework is intentionally combined with a second ion‐conducting phase (usually a polyether/polar polymer, a poly (ionic liquid), or a confined salt‐rich phase) to simultaneously optimize ion solvation/dissociation, pathway continuity, and mechanical integrity. In contrast to COF‐only pressed bodies (in which particle contacts and voids can dominate macroscopic resistance), hybrids use the COF as a nanoscale template that reshapes the local dynamics of the guest phase and concentrates Li^+^‐coordinating functionalities within continuous porous networks.

From a structure–activity perspective, the “hybrid” label is only meaningful when the second phase is not merely a passive binder but an active participant in ion transport. Mechanistically, hybridization can (i) inhibit crystallisation of polymer segments through nanoconfinement, (ii) enhance the number of dissociated charge carriers through coordination of anions/cations on polar pore walls, (iii) create percolating conduction corridors through bridging of particle‐contact regions by an infiltrated polymer/salt network, and (iv) create hierarchical transport where fast intra‐channel hopping is coupled to short‐range segmental relaxation within the polymeric phase. Recent demonstrations, including covalently tethered oligoether chains, COF‐filled polymer electrolytes, and confined eutectic‐like phases, collectively suggest that the COF scaffold can be utilized as a “structure director” for the surrounding ion‐conducting matrix rather than a simple filler [50, 51].

A recurring mechanistic ambiguity in hybrid COF electrolytes is pathway dominance: depending on the pore filling and percolation, Li^+^ current may essentially flow through (i) COF corridors (particularly in salt‐in‐COF or single‐ion frameworks), (ii) the polymer/plastic‐crystal phase (in framework‐in‐polymer composites), or (iii) mixed pathways with the COF surfaces functioning as salt dissociation/anion binding sites and the secondary phase taking up most of the current. Distinguishing between these scenarios requires controls (polymer‐only, COF‐only, and blocked‐pore analogs) and spatially resolved probes (e.g., solid‐state NMR, thickness‐scaled impedance, and anisotropic transport measurements), rather than attributing conductivity gains solely to “ion highways.”

### 5.1 Hybridization Archetypes and Design Logic

Hybrid COF electrolytes can be divided into three archetypes that correspond neatly to structural levers discussed in the previous sections: (1) polymer‐in‐framework designs, in which solvating chains are covalently grafted to the pore walls or accumulated in the channels of a crystalline COF; (2) framework‐in‐polymer composites, in which COF crystallites (often functionalized) are dispersed in a polymer electrolyte to modulate salt dissociation, polymer crystallinity and tortuosity; and (3) phase‐confined hybrids, wherein a salt‐rich ion‐conductive phase (e.g., ionic liquid/eutectic‐like complexes or plastic‐crystal‐like domains) is immobilized within a charged or zwitterionic COF channel system to deliver liquid‐like local mobility while retaining a solid form factor.

Across these archetypes, a common pattern emerges: the best hybrids simultaneously improve ionic conductivity and the Li^+^ transference number, indicating a transition from coupled cation/anion motion to more selective Li^+^ motion. This selectivity can be due to anion‐trapping sites on the COF walls (cationic or Zwitterionic frameworks) or to “crowding” in the ether‐rich nanochannels, which sterically and electrostatically restrict bulky anions while retaining Li^+^‐ether coordination.

### 5.2 Polymer Tethering and Polymer Accumulation Inside COF Channels

Polymer‐in‐framework approaches are conceptually appealing, as they create a solvating environment directly within a crystalline framework that minimizes macroscopic contributions to resistance arising from particle contacts and allows well‐defined ion coordination geometries. A prototypical demonstration is the hydrazone‐linked COF‐PEO_‐x_ series reported by Horike and co‐workers, in which oligo (ethylene oxide) chains (x = 3, 6, 9) are incorporated during COF formation, creating a periodic pore environment with solvating ether segments. Figure 14 shows that changing the PEO chain length adjusts the density of ether functionalities within the crystalline COF channels. Solid‐state NMR and thermal analyses showed that the confined PEO chains exhibit highly dynamic, glassy‐like behavior, and LiTFSI doping gave a solid‐state Li^+^ conductivity of 1.33 × 10^−3^ S cm^−1^ at 200°C, with the transport response sensitive to chain length and polymer dynamics [51].

**FIGURE 14:**
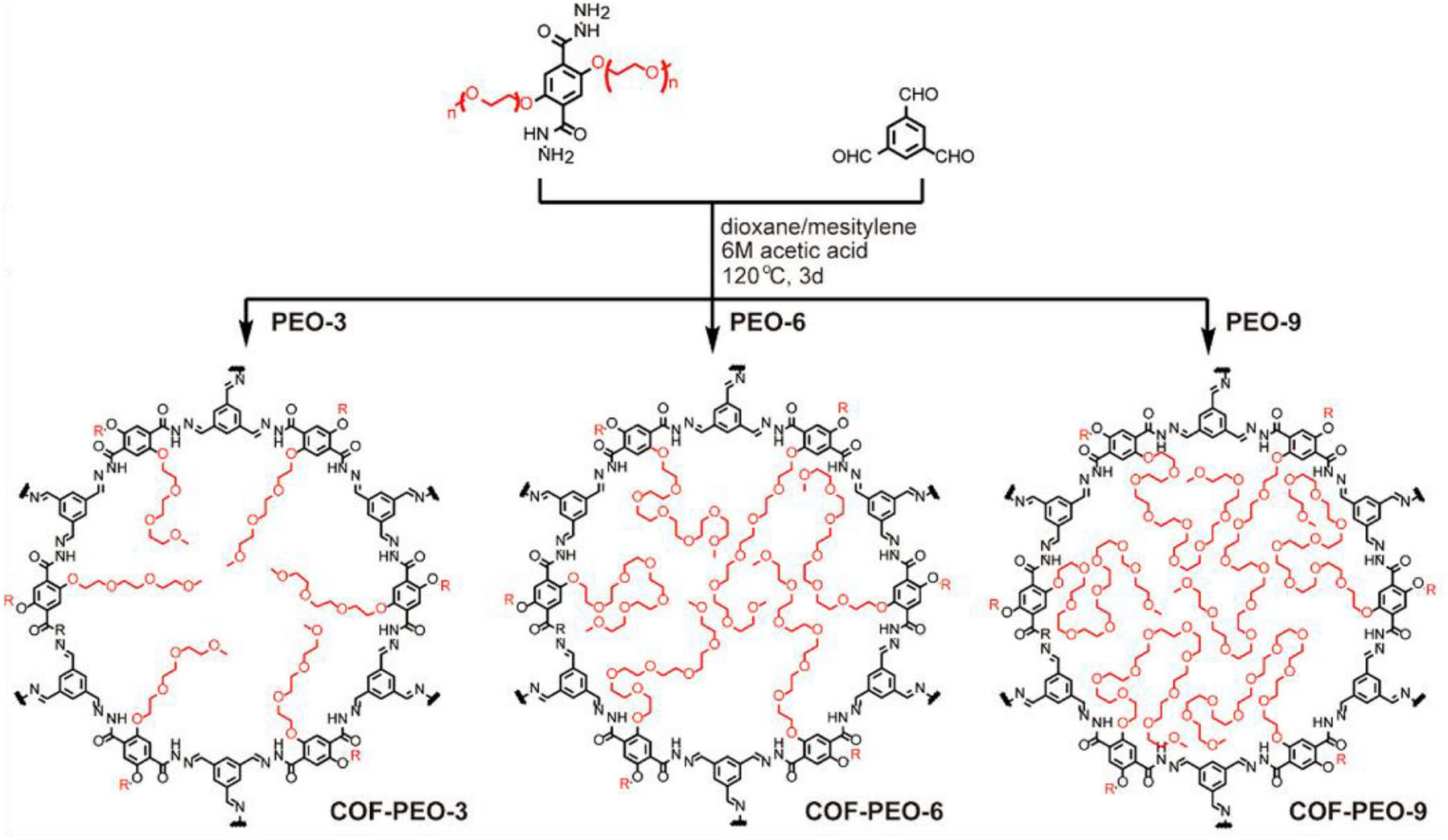
Polymer‐in‐framework hybridization concept: synthesis of COF‐PEO‐x (x = 3, 6, 9) showing ethylene oxide chains incorporated into a crystalline COF to create ether‐lined nanochannels. Reproduced with permission [49].

A key design implication of such tethered systems is that “polymer content” must be treated as a topological variable, since longer or more crowded ether chains increase the density of coordinating sites but can also reduce free volume and introduce local kinetic bottlenecks. This balance is clearly reflected in the branched PEG‐functionalized COFs, where Wang et al. proposed that the crowded nanochannels can limit anion displacement, while preserving coordination of Li^+^‐ethers [49]. After LiTFSI incorporation, the most crowded branched‐PEG variant exhibited an ionic conductivity of 1.5 × 10^−3^ S cm^−1^ at 200°C. It gave a higher Li^+^ transference number (0.30 at 100°C) than a less‐crowded analog (0.18 at 100°C), supporting the hypothesis that steric/electrostatic confinement can bias transport toward Li^+^ motion. At high tether densities or excessive polymer amounts, the pore centers can be crowded/occluded by ether chains, thereby decreasing the effective cross‐section and increasing steric friction. As a result, σ may exhibit a non‐monotonic dependence on “polymer content” in polymer‐in‐framework COFs.

Extending polymer‐in‐framework logic to 3‐D topologies, Wu et al. assembled PEG‐functionalized 3‐D COFs with multidirectional channels, aiming to minimize particle‐contact resistance and achieve (more) isotropic percolation in pelletized or membrane‐like solids [56]. The best‐performing 3D‐COF‐PEG6‐Li system achieved an ionic conductivity of 3.6 × 10^−4^ S cm^−1^ at 260°C, with a reported Li^+^ transference number of about 0.22, demonstrating both the high‐temperature potential and the accessibility challenge of selective ether‐based COF polyelectrolytes. Collectively, these studies highlight that polymer tethering is most effective when the COF topology and tether density are co‐designed, ensuring that solvating groups are abundant yet do not collapse effective free volume or disrupt long‐range channel coherence.

### 5.3 COF Crystallites as Functional Fillers in Polymer Electrolytes

Framework‐in‐polymer composites are the most widely explored hybrid class, as they maintain the ease of manufacture of polymer electrolytes while introducing COF‐derived order, porosity, and tailored Lewis basic/Lewis acid sites. In these systems, COFs may facilitate salt dissociation and increase the fraction of amorphous polymer segments near the filler surface, thereby increasing both charge‐carrier concentration and segment‐assisted hopping. Representative PEO‐based composites incorporating neutral or cationic COFs have demonstrated that small COF loadings can simultaneously reduce polymer crystallinity while increasing the number of solvating/anion‐binding sites, leading to measurable improvements in room‐temperature conductivity and cycling stability.

A mechanistically instructive case is the fibrous, carbonyl‐rich COF (TPBD) that was combined with LiPF_6_ and PEO, which formed a continuous conducting network with ionic conductivities of 0.543 mS cm^−1^ at 25°C and 3.23 mS cm^−1^ at 70°C (activation energy ≈ 0.37 eV) [30]. Importantly for structure–activity reasoning, it was reported in the same study that TPBD‐LiPF_6_ (without PEO) had significantly lower conductivity (0.00314 mS cm^−1^ at 25°C), suggesting that the polymer phase in the hybrid is not just a binder, but an essential bridge that links the ion‐conducting domains from COFs into a macroscopic percolation network.

A uniquely high COF‐polymer synergy is seen in COF‐5@LiI/PEO composite electrolytes for all‐organic solid‐state cells, where a LiI‐impregnated COF‐5 additive led to measurable ion conduction independently (6.0 × 10^−5^ S cm^−1^ at 19°C; 3.6 × 10^−3^ S cm^−1^ at 70°C) and stable cycling in a TCNQ‐based lithium metal battery configuration. Although the chemistry and operating window differ from those of carbonate‐salt polymer electrolytes, this work demonstrates a generalizable hybrid principle: embedding a salt‐bearing COF phase within a polymer matrix can decouple the salt's solubility requirements from those of the electroactive components while maintaining a fully solid formulation.

Taken together, composite studies suggest that COF fillers provide the greatest benefit if the pore‐wall chemistry is optimized to participate actively in the salt/solvent‐free coordination environment, for example, carbonyl or cationic sites that facilitate dissociation of common salts, and if the composite microstructure provides uniform dispersion and short distances for polymer bridging between adjacent COF particles. Under such conditions, the effective ion highway becomes hierarchical, in the sense that intra‐channel conduction within the COF favors low‐tortuosity segments. In contrast, the polymer‐rich regions facilitate local relaxations that allow ions to traverse the interparticle landscape without incurring severe energetic penalties.

### 5.4 In Situ Polymerization and Junction Engineering in COF‐Polymer Hybrids

While ex situ blending is scalable, it often fails to provide complete pore infiltration and intimate contact between the COF and the polymer at the nanoscale. In situ polymerization is an elegant solution to this problem, using the COF as a confined reactor or catalyst platform, in which polymer growth occurs within or around the framework. The pores are filled, and particles are bridged from the beginning. This approach can minimize the number of transport dead zones and create dense, continuous conduction corridors without compromising the long‐range order of the COF scaffold.

Meng et al. demonstrated the use of COF‐induced ring‐opening copolymerization to in situ form solid polymer electrolytes, yielding polymer@TpPa‐COOLi hybrids in which the lithium carboxylate sites are incorporated into the COF and participate in ion transport. In the reported series, the introduction of COFs into the polymer matrix increased the lithium‐ion transference number from 0.66 to 0.85 for polymer@TpPa‐COOLi, thereby indicating a strong suppression of anion mobility relative to Li^+^ ion motion. Conductivity trends further illustrated the structural role of the COF: at 60°C, polymer@TpPa‐COOLi exhibited a conductivity of 5.62 × 10^−5^ S cm^−1^. At the same time, the COF‐free polymer and a control hybrid without the lithium‐carboxylate motif exhibited lower values in the same temperature range.

Conceptually speaking, in situ polymerization is in good agreement with the topology design of COFs because the framework can be designed to (i) expose the catalytic or initiating sites on the pore walls, (ii) set confinement length scales to control the packing and crystallisation tendency of polymer chains and (iii) enforce a pathway on the formed polymer phase that has a single dimension or is three‐dimensionally connected. In this sense, in situ growth transforms the COF from an additive to an active “polymerization scaffold,” enabling co‐optimization of ion‐solvation chemistry and pathway geometry, a feat difficult to achieve with post‐mixing routes.

### 5.5 Confined Salt‐Rich Phases and Eutectic‐Like Hybrids in COF Channels

A complementary direction of hybridization is to place a salt‐rich, locally mobile phase within a rigid COF host to achieve a balance between liquid‐like short‐range dynamics and a macroscopically solid framework. Zwitterionic and ionic COFs are particularly appealing hosts for this approach, as internal charge separation can trap counterions and stabilize concentrated ionic domains within the ordered channels.

In an illustrative example, by complexation with Pyrrol‐FSI and Li salts, the researchers (Lee et al.) were able to incorporate a eutectic‐like conductive phase into a Zwitterionic PSZ‐COF, which delivered ionic conductivity of 1.27 × 10^−4^ S cm^−1^ at room temperature, along with a high Li‐ion transference number of 0.62 (activation energy ≈ 0.151 eV). Notably, the room‐temperature conductivity of the pristine PSZ‐COF without the eutectic‐like complex was lower (7.28 × 10^−5^ S cm^−1^) and the activation barrier higher, providing support for the view that the confined conductive phase plays a major role in enhancing Li^+^ migration while the semiconductive framework biases away the motion of the anionic framework.

Related “phase‐confined” logic is also apparent in ionic COF composites incorporating polymerized ionic segments or other ionically active binders, where the guest phase both acts as a mechanical glue and an auxiliary conduction medium, and the COF channel system controls directional order and suppresses anion drift. These results all point to a design space in which the guest phase is optimized for local mobility (low activation energy) and the framework is optimized for selectivity (anion immobilization and channel continuity).

## 6 Challenges and Research Gaps

Prior to addressing individual challenges, it is important to contextualize COF electrolytes relative to leading inorganic and polymer solid‐electrolyte families and to consolidate the quantitative metrics of representative COF systems discussed in Sections 4 and 5. Table 4 presents a class‐level, side‐by‐side comparison of COF‐based electrolytes with sulfide, garnet, and halide inorganic conductors, as well as with dry polymer electrolytes. Table 3 compiles, for leading COF systems, the structural descriptors (pore size, framework dimensionality, and charge) alongside σ, E_a_, and t_Li+_, together with the electrolyte form in which each value was measured. Two key observations frame the subsequent discussion of challenges. First, COF electrolytes now span the conductivity range between dry polymers and the most effective inorganic conductors, while providing among the highest lithium‐ion selectivity of any processable electrolyte class. Second, membrane thickness and σ_eff_ are systematically under‐reported, which currently impedes areal‐resistance benchmarking across the field. The sparsity of these entries in Table 3 quantitatively illustrates the reporting gap addressed in Section 6.5.

**TABLE 4:** Benchmark comparison of representative COF‐based electrolytes with major classes of solid electrolytes.

Property	Sulfides (e.g., argyrodite, LGPS) [12]	Garnets (LLZO) [11, 80]	Halides (Li_3_MCl_6_) [81]	Dry polymers (PEO‐LiTFSI) [82]	COFs [83, 84]
σ at RT (S cm^−1^)	10^−3^–10^−2^	10^−4^–10^−3^	10^−4^–10^−3^	10^−6^–10^−5^	10^−6^–10^−2^ (up to ∼10^−3^ in hybrids)
t_Li+_	≈1	≈1	≈1	0.1–0.4	0.3–0.97 (highest in anionic/single‐ion COFs)
E_a_ (eV)	0.2–0.35	0.3–0.45	0.3–0.45	0.5–1.0	0.1–0.5 (system‐dependent)
Stability	Narrow intrinsic window; H_2_S release on moisture exposure	Stable vs. Li metal; surface Li_2_CO_3_ raises interfacial resistance	Good oxidative stability; unstable vs. Li metal	Oxidation above ∼4 V limits high‐voltage cathodes	Linkage‐dependent; imines reducible at Li; windows rarely validated at practical loadings
Key limitation	Moisture and electrochemical instability	Rigid interfaces; dendrite penetration along grain boundaries	Cost; Li‐metal incompatibility	Low RT conductivity and tLi^+^	σ_eff_ gap vs. intrinsic transport; free‐standing membranes immature

### 6.1 Room Temperature Is “Promising on Paper, Difficult in Practice”

COFs offer ordered nanochannels and chemically programmable coordination environments that, in principle, should enable rapid Li^+^ migration. In practice, however, many COF‐based solid electrolytes still struggle to consistently deliver high ionic conductivities at/near room temperature unless plasticizers, high fractions of mobile guest phases, or polymer/gel components are used. This limitation is closely tied to the dominant mode of ion transport within COF pores: depending on the pore filling, solvation structure, and Li^+^‐framework interactions, ion transport can occur via site‐to‐site hopping between confined coordination motifs or via vehicular motion of solvated Li^+^ complexes. These mechanisms have distinct activation penalties, and their relative contributions often vary with temperature, salt loading, and the nature of the guest phase, leading to substantial variability in σ and E_a_ across otherwise similar COF platforms [85].

### 6.2 The σ‐Ea‐t_Li+_ Coupling Is not yet Decoupled by Design

A lasting challenge is that strategies that aim at improving one transport metric often compromise another. Increasing the density of coordinating donor sites promotes salt dissociation and sometimes increases σ; however, excessive Li^+^ coordination can raise the migration barrier and E_a,_ ultimately limiting performance at room temperature. Likewise, the introduction of fixed charges (ionic COFs) may improve Li^+^ selectivity and increase apparent transference behavior. Still, it may lead to reduced overall mobility if ion aggregates persist, if counterion interactions remain high, or if Li^+^ becomes too “trapped” in a highly interactive pore environment [85]. The field, therefore, still lacks transferable, descriptor‐based design rules for balancing donor strength, donor density, and framework charge density to simultaneously maximize σ and minimize E_a_ while providing meaningful Li^+^‐selective transport.

### 6.3 Crystallinity Is Supportive of Pathways, but Microstructure Often Determines σ_eff_

In ideal COF crystals, continuous channels can act as well‐defined conduction paths. In actual membranes and in pellets, however, the ion transport is often determined by microstructural factors‐crystallite size, misalignment, porosity/voids, and, particularly, grain boundaries, which provide additional resistance and tortuousness. As a result, the experimentally observed effective conductivity (σ_eff_) may deviate substantially from the intrinsic channel‐level transport expected solely from the framework structure. Recent reviews highlight the importance of orientation control and microstructural optimization to minimize the grain‐boundary penalty and translate intrinsic “Ion Highway” transport into practical σ_eff_ values [86].

### 6.4 Defects and “Doping” Under‐Quantified: Beneficial, Detrimental, or Simply Uncontrolled?

Defects can, in principle, be exploited to tune ion transport by generating additional coordination sites, altering free volume, or locally changing electrostatics. Conversely, defects may introduce trap states, disrupt long‐range order, and fragment long percolating pathways, thereby increasing E_a_ and reducing σ. Yet, in many COF electrolyte studies, the defects are discussed only qualitatively (e.g., peak broadening or microscopy images) without quantitative characterization or correlational analyses, such as defect density versus E_a_ change or defects/disorder versus percolation breakdown. Establishing standardised metrics for disorders (and reporting them alongside transport data) is thus a crucial gap in the framework for transitioning from empirical optimization to predictive design.

### 6.5 Channel Alignment and Thickness Effects: Anisotropic Transport Is Expected, and Seldom Demonstrated Rigorously

A fundamental promise of COFs is directional ion transport, enabled by aligned channels and ordered pore architectures. Nevertheless, most electrochemical measurements are reported as σ values across the thickness for partially heterogeneous or unverified channel‐alignment membranes. In such cases, orientation, thickness, and macroscopic texturing can be the dominant factors governing measured resistance and apparent E_a_, obscuring intrinsic structure–transport relationships. Encouragingly, scalable fabrication routes for COF‐based membrane electrolytes are emerging, aiming to incorporate crystallinity and channel directionality into robust, testable architectures. However, the community still requires systematic studies relating alignment state and thickness to anisotropic transport metrics and device‐relevant σ_eff_ [35].

### 6.6 Li^+^ Transference Assessments (T_Li+_) Are Prone to Methodological Artifacts

Since this review emphasizes correlations between structure and function, the t_Li+_ needs to be interpreted with caution. The commonly used Bruce‐Vincent polarisation method has the potential to produce artificially high t_Li+_ values when other assumptions about its operation (e.g., ideal behavior, stable electrode contacts, absence of concentration polarisation artifacts) are not met—exactly the problem pointed out in the wider battery literature. In addition, modern transport frameworks demonstrate that the “true” transference behavior can differ markedly from simplified estimates, especially in correlated or concentrated systems. Consequently, high t_Li+_ claims should be accompanied by complete methodological details (polarisation conditions, impedance before/after, steady‐state criteria) and, if possible, cross‐validation or consistency checks across concentration/temperature [87].

### 6.7 Mechanistic Understanding Still Trails Materials Innovation

Many reports on COF electrolytes show enhanced σ or t_Li+_ after functionalization, guest incorporation, or charge engineering of the framework; however, the mechanistic rationale is usually inferred rather than directly demonstrated. The fact that ion motion in COFs is sensitive to pore filling, solvation structure, and whether migration proceeds predominantly by hopping or by vehicular transport is a feature that requires combining experimental probes and modeling to resolve. As emphasised in the broader ion‐transport literature, additional, enhanced mechanistic validation (correlating local coordination and migration barriers with observed σ(T) and E_a_) is required to bridge the gap between empirical materials discovery and predictive “Ion Highway” design principles [35, 88, 89].

### 6.8 Interfacial Stability, SEI Evolution, and Dendrite Suppression Remain Underexplored Relative to Bulk Transport

Most studies on COF electrolytes, including those reviewed in Sections 4 and 5, focus on bulk ion conduction within the framework. In contrast, the interfaces that ultimately determine cell‐level performance have received significantly less attention. At the lithium‐metal anode, the chemical stability of the linkage is a major concern. Imine and other polarizable linkages may be reduced upon contact with freshly plated lithium, and confined LiTFSI or plasticizer phases can decompose to form an interphase. The composition of this interphase (LiF, Li_3_N, organic fragments) is rarely characterized with the rigor routinely applied to inorganic and liquid systems [47]. Recent advances suggest that COF‐specific interphase strategies are emerging. Periodic lithiophilic sites on COF pore walls can homogenize interfacial Li^+^ flux and promote compact, dendrite‐free deposition [68]. Furthermore, some solution‐processable lithiophilic COFs have been shown to serve as artificial solid–electrolyte interphase (SEI) layers that stabilize lithium plating and stripping during extended cycling [90]. Mechanistically, dendrite suppression by COF electrolytes is expected to result from a combination of uniform Li^+^ flux imposed by the periodic channel array, high tLi^+^ enabled by anion immobilization (which suppresses space‐charge and concentration polarization that trigger dendrite nucleation), and pore‐size sieving that restricts anion transport while permitting Li^+^. However, direct evidence that disentangles these contributions, such as Li/COF/Li critical‐current‐density measurements and operando visualization, remains limited.

The cathode interface has received even less investigation. Integrating COF electrolytes with high‐voltage, nickel‐rich layered oxide cathodes requires oxidative stability of at least 4.3 V vs. Li/Li^+^. However, the electrochemical stability windows for COFs are typically determined using sweep voltammetry on inert electrodes [91], a method that often overestimates practical oxidative limits. Interfacial side reactions with the reactive delithiated cathode surface, transition‐metal crosstalk, and contact loss during cathode breathing remain largely unexplored in COF systems. Nonetheless, the conformability of COF‐polymer hybrids and the availability of thin, solution‐processable COF coatings suggest promising strategies for engineering cathode–electrolyte interphases.

Future COF electrolyte studies should, at a minimum, report the evolution of interfacial impedance during Li/electrolyte/Li symmetric cycling, critical current density, post‐interphase characterization (such as XPS depth profiling or cryo‐electron microscopy), and, for full cells, cycling data with practically relevant cathodes (such as LiFePO4 and Ni‐rich layered oxides) and controlled lithium excess [92]. These data represent the interfacial counterpart to the transport‐reporting toolkit proposed in Section 7.4. and are essential for assessing whether ion‐highway designs remain effective when in contact with real electrodes.

## 7 Future Perspectives—Design Rules for Ion Highways for Next‐Generation Energy Storage

The future development of solid electrolytes based on COFs is less likely to be driven by isolated “best‐performing” demonstrations and more by predictive Ion Highway design: quantitative links that connect framework descriptors (topology, channel continuity, pore‐wall fields, and microstructure) to transport signatures (σ, E_a_, t_Li+_, anisotropy, and σ_eff_). The preceding sections demonstrate that high performance results from the co‐optimization of connectivity, coordination, crystallographic coherence, and cooperative hybridization, rather than the independent tuning of each of these factors. The next stage of the field is therefore a move from materials‐by‐example to descriptors to mechanisms to metric maps that are transferable across COF families and device formats [93].

### 7.1 A Four‐Lever Roadmap for Ion Highway Design

Throughout Topology and Channel Architecture, Chemical Functionality of Pore Walls, Crystallinity and Hybrid COFs, a consistent design story can be observed: (i) the framework to define a continuous pathway, (ii) the channel wall to define the Li^+^ energy landscape, (iii) crystalline coherence to determine whether the intrinsic pathway will survive at practical thickness, and (iv) a cooperative guest or hybrid phase to select the dominant transport mode under confinement. Translating this story into design rules helps clarify what should be optimized and what performance signature should be delivered.

**Design Rule 1**: Geometry defined by connectivity. Cause: net topology, stacking registry, and channel alignment with a low‐tortuosity axial pathway. Effect: decreased bottleneck density, decreased effective diffusion length. Performance signature: higher σ_eff_ at fixed thickness and clearer anisotropic transport.
**Design Rule 2**: Coordination Characterizes the Energy Landscape. Cause: periodic donor/charge motifs on the pore wall tune Li^+^ binding and handoff frequency. Effect: reduced migration barriers and no deep trapping wells. Performance signature: simultaneous decrease of E_a_ with maintenance or increase of σ and t_Li+_.
**Design Rule 3**: The crystalline coherence maintains pathway fidelity. Cause: crystallinity, domain size, defect control—maintain the long‐range periodicity. Effect: fewer boundary crossings and less resistance due to morphology. Performance signature: σ_eff_ is close to intrinsic channel transport and varies predictably with thickness.
**Design Rule 4**: Cooperative hybridization determines the mode of transport. Cause: confined polymers, ionic segments, gels, or plastic‐crystal guests determine pore occupancy and local segmental mobility. Effect: controlled crossover between hopping and vehicular transportation in the same channel scaffold. Performance signature: improved near‐room‐temperature σ without loss of channel selectivity and mechanical form.

The opportunity is that these descriptors can be translated into design maps that can be used to predict not just σ, but the full σ‐E_a_‐t_Li+_ coupling. In particular, design rules should dictate how donor strength/density and charge spacing can be used to increase carrier density without inducing deep binding wells that increase E_a_, and how topology and alignment convert intrinsic channel transport into σ_eff_ at practical thicknesses.

### 7.2 Mechanistic Validation that Verifies the Highway

Mechanistic understanding still lags behind materials innovation, and future work should focus on “transport‐proof” datasets that make direct links between local coordination dynamics and macroscopic σ(T). A useful standard is to couple the temperature‐dependent conductivity and the extracted E_a_ with at least one direct probe of Li^+^ motion or coordination exchange (e.g., solid‐state NMR) and a validated simulation model. Molecular dynamics simulations within confined COF channels already show the rationalization of liquid‐like 1D diffusion via probability‐density maps and coordination rearrangements in the channels when pores are suitably filled with appropriate embodiments. At the same time, DFT‐level barrier calculations can determine whether axial migration is favored by the desired wall chemistry and stacking registry.

To speed convergence, the community would benefit from benchmark families in which only a single variable is altered at a time (e.g., donor density series at fixed topology; stacking control series at fixed chemistry; or pore size series at fixed charge spacing) so that cause‐and‐effect relationships can be cleanly extracted. Such controlled libraries will also allow meaningful validation of computational screening, in which predicted diffusion barriers and favored pathways are compared with measured σ(T), E_a_, and anisotropy [94, 95].

### 7.3 Microstructure and Thickness Scaling as Primary Performance Targets

Even in the case of high intrinsic channel transport, the reality is determined by σ_eff_ and the areal resistance (RA = thickness/σ_eff_). Accordingly, microstructure‐aware design needs to be treated as a first‐class lever and not a processing afterthought. Reports should routinely include membrane‐thickness series, alignment measurements, and anisotropic conductivity (in‐plane vs. through‐plane) to determine whether the designed channels are continuous ion highways across the full electrolyte thickness [96, 97].

Future materials should therefore be judged not just by peaks but by the robustness of σ_eff_ under realistic thickness and densification. Topological strategies that maintain axial continuity (stacking control in 2D COFs and mechanically continuous 3D nets) should be combined with fabrication routes that offer low boundary resistance and low void fractions, so that σeff approaches intrinsic transport rather than being dominated by morphological losses [96].

To translate these considerations into quantitative targets, cell‐level benchmarks for COF electrolyte membranes are established based on published benchmarking analyses of solid‐state batteries [98, 99, 100]. The electrolyte layer directly affects both areal resistance and the gravimetric or volumetric energy of a cell. Therefore, free‐standing COF membranes should be processed to thicknesses well below 100 µm, with 50 µm representing a more practical objective. A 50 µm‐thick membrane with σ_eff_ =  1.0 × 10^−4^S cm^−^^1^ would exhibit an area‐specific resistance of 50 Ω cm^2^. Reducing the resistance to 20 and 10 Ω cm^2^ would require σ_eff_values of approximately 2.5 × 10^−4^and 5.0 × 10^−4^S cm^−^^1^, respectively [98]. At the lithium interface, symmetric Li/electrolyte/Li cells should support current densities exceeding 1 mA cm^−2^, and ideally 3 mA cm^−2^ or higher for fast charging, while cycling areal capacities of 1–3 mAh cm^−2^ [100]. Device‐level validation should be based on full cells assembled with realistic chemistries, such as Ni‐rich layered oxide cathodes operating at 4.3 V versus Li/Li^+^ under practical loading, thin lithium‐metal anodes, and capacity retention demonstrated over hundreds of cycles rather than only a few tens [98]. When evaluated against these benchmarks, the gap between record intrinsic conductivities and a deployable COF membrane becomes a quantifiable engineering challenge rather than a qualitative distinction.

### 7.4 A Measurement and Reporting Toolkit, which Allows for Reliable Design Rules

Measurement inconsistency stands as a fundamental barrier to establishing reliable, predictive design principles. Two priorities, in our view, offer the greatest potential to advance the field: first, adopting standardized protocols for measuring and reporting the essential dataset outlined below, and second, systematically synthesizing benchmark COF series in which only one structural parameter is varied at a time. Future studies should be conducted to standardize the measurement and reporting of σ, E_a_, and t_Li+_; cell geometry; temperature window; polarisation condition; steady‐state criteria; and impedance before/after polarisation. Because t_Li+_ estimates can be sensitive to methodological assumptions and correlated transport, high t_Li+_ claims should be accompanied by consistency checks between concentration and temperature and, where possible, cross‐validation using complementary analysis frameworks. A useful community minimum dataset would include: (1) structural descriptors (pore size distribution, interlayer spacing/stacking registry, texture/orientation and porosity/density) (2) composition and occupancy (salt identity/loading; guest/polymer fraction) (3) σ(T) with thickness and cell geometry plus extracted E_a_ (4) t_Li+_ with the measurement method and uncertainty (5) electrochemical stability window and Li‐metal contact stability (6) mechanical/processing descriptors (modulus/compressibility; areal resistance at practical thickness) [97, 101, 102, 103].

### 7.5 Transferability and Data‐Driven Acceleration

Finally, Ion Highways should be considered a transport‐only platform and not just a Li‐only solution. The same design logic, continuous channels, programmable donor fields, controlled confinement, and microstructure‐aligned pathways should be transferable to Na^+^, K^+^, and other charge carriers after re‐optimizing pore dimensions, polarity, and coordination strength for their distinct solvation requirements. This expectation is reinforced by parallel advances in organic electrode materials for aqueous monovalent‐ion batteries, which similarly suggest that design logic independent of a specific charge carrier can be applied broadly across organic energy‐storage systems [104]. Establishing open datasets of descriptor‐metric pairings for multiple ions and salts would help realize data‐driven screening and advance empirical discovery into a predictive electrolyte‐engineering workflow [94, 95, 105]

Overall, the most impactful next step is to construct quantitative descriptor‐to‐mechanism‐to‐metric maps that jointly optimize σ, E_a_, t_Li+_, anisotropy, and σ_eff_. When combined with microstructure and occupancy descriptors, COF electrolytes can be designed as predictable, device‐ready ion highways rather than as empirically tuned materials.

## 8 Conclusion

Covalent organic frameworks have evolved from porous hosts to designable transport architectures for solid‐state lithium batteries. Across the literature surveyed here, the best performance enhancements are observed when COFs are regarded as “ion‐highway” scaffolds whose function is determined by the coupled optimization of connectivity (topology and channel continuity), coordination (pore‐wall donor chemistry and framework charge), coherence (stacking registry, crystallinity, and texture), and hybridization (controlled pore filling with mobile conducting media). This cause‐to‐effect‐to‐performance framing offers a practical path to rationalize the observed fact that some COF electrolytes exhibit simultaneously high ionic conductivity and high lithium‐ion transference. In contrast, others exhibit promising intrinsic transport that is not translated into device‐relevant effective conductivity.

First, connectivity determines whether Li^+^ has a percolating corridor or a fragmented pathway. Layered 2D COFs can form low‐tortuosity axial channels in well‐registered domains, while 3D nets minimize penalties associated with misorientation but entail trade‐offs in interpenetration and aperture narrowing. Second, coordination determines the Li^+^ energy landscape. Neutral lithiophilic donors can mimic solvation and induce fast handoff, but overbinding raises activation barriers; fixed‐charge frameworks (anionic, cationic, or even mixed‐charge or zwitterionic) can bias transport in favor of Li^+^ (immobilizing or electrostatically confining counter‐anions), provided pore accessibility and free volume remain. Third, coherence is the bridge between molecular design and macroscopic performance: stacking disorder, small crystallites, and a grain‐boundary‐rich microstructure may become the leading causes of resistance, widening the gap between intrinsic σ and membrane‐scale σ_eff_.

Hybrid COF electrolytes offer a pragmatic approach to combining the features of ordered corridors with locally mobile media, particularly when slow coordination rearrangements limit operation at room temperature in rigid frameworks. However, hybridization must be engineered rather than appended: pore‐filling fraction, tether density, and guest‐phase chemistry must be tuned to prevent channel congestion and maintain continuous conduction across the electrolyte thickness. In this context, peak conductivity is not a sufficient performance metric; areal resistance and thickness scaling (RA = thickness/σ_eff_), along with t_Li+_ and temperature‐dependent activation energies, better characterize the practical value of a given “ion‐highway” design.

Looking to the future, a quantitative descriptor for transport maps based on standardized reporting would do the most for the field. At the very least, studies should report pore‐size distributions and interlayer spacing/registry; concentrations of functional groups (donor and fixed‐charge sites), pore‐filling fractions, and salt stoichiometries; alignment/texture descriptors for membranes; and σ(T) with extracted E_a_. Mechanistic validation should complement these metrics with at least one direct probe of Li^+^ motion or coordination exchange, along with a validated computational/transport model, thereby enabling the distinction between hopping‐dominated and guest‐assisted regimes. With these practices, COF electrolytes can be advanced from materials‐by‐example to predictive ion‐conducting pathway engineering, accelerating the realization of safe, high‐energy solid‐state lithium batteries.

## Author Contributions

**Rajesh Dhanushkotti**: conceptualization, Writing – original draft, Writing – review and editing. **Ningaraju G.N**. and **Karthik C.S**.: data curation, writing – review and editing. **Karthik V**, **Anil Kumar M R**, **Jitendrasingh Rajpurohit**, and **Jeremy I.G Dawkins**: writing – review and editing. **Karim Zaghib**: conceptualization, data curation, supervision, writing – review and editing.

## Conflicts of Interest

The authors declare no conflicts of interest.

## Data Availability

No data were used for the research described in the article.
